# Ct-OATP1B3 promotes high-grade serous ovarian cancer metastasis by regulation of fatty acid beta-oxidation and oxidative phosphorylation

**DOI:** 10.1038/s41419-022-05014-1

**Published:** 2022-06-18

**Authors:** Yutang Huang, Yan Du, Yujie Zheng, Chunjie Wen, Hecun Zou, Jiafeng Huang, Honghao Zhou, Hongbo Zhao, Lanxiang Wu

**Affiliations:** 1 Institute of Life Sciences, Chongqing Medical University, Chongqing, 400016 China; 2 Obstetrics and Gynecology Hospital, Fudan University, Shanghai, 200011 China; 3 Department of Obstetrics and Gynecology of Shanghai Medical School, Fudan University, Shanghai, 200032 China; 4 Pharmacogenetics Research Institute, Institute of Clinical Pharmacology, Central South University, Changsha, 410078 China; 5 Shanghai Key Laboratory of Female Reproductive Endocrine Related Diseases, Shanghai, 200011 China; 6 Molecular Medicine Diagnostic and Testing Center, Chongqing Medical University, Chongqing, 400016 China

**Keywords:** Metastasis, Metabolomics

## Abstract

High-grade serous ovarian cancer (HGSOC) is the most lethal gynecologic malignancy mainly due to its extensive metastasis. Cancer-type organic anion transporting polypeptide 1B3 (Ct-OATP1B3), a newly discovered splice variant of *solute carrier organic anion transporter family member 1B3 (SLCO1B3)*, has been reported to be overexpressed in several types of cancer. However, the biological function of Ct-OATP1B3 remains largely unknown. Here, we reveal that Ct-OATP1B3 is overexpressed in HGSOC and promotes the metastasis of HGSOC in vivo and in vitro. Mechanically, Ct-OATP1B3 directly interacts with insulin-like growth factor 2 mRNA-binding protein 2 (IGF2BP2), an RNA-binding protein, which results in enhancement of the mRNA stability and expression of carnitine palmitoyltransferase 1A (CPT1A) and NADH:Ubiquinone Oxidoreductase Subunit A2 (NDUFA2), leading to increased mitochondrial fatty acid beta-oxidation (FAO) and oxidative phosphorylation (OXPHOS) activities. The increased FAO and OXPHOS activities further facilitate adenosine triphosphate (ATP) production and cellular lamellipodia formation, which is the initial step in the processes of tumor cell migration and invasion. Taken together, our study provides an insight into the function and underlying mechanism of Ct-OATP1B3 in HGSOC metastasis, and highlights Ct-OATP1B3 as a novel prognostic marker as well as therapeutic target in HGSOC.

## Introduction

High-grade serous ovarian cancer (HGSOC), the most common form of ovarian cancer, is known for its high rate of metastasis and accounts for 70–80% of ovarian cancer deaths [1]. Due to the lack of effective early screening options and the paucity of specific symptoms, the majority of cases are diagnosed at an advanced stage with distant metastases. As a result, the effectiveness of debulking surgery or chemotherapy is limited, and the 5-year overall survival rate is <30% [2]. Although HGSOC metastasis has been extensively investigated, the underlying molecular mechanisms are still not fully understood.

Cancer-type OATP1B3 (Ct-OATP1B3) is a newly identified splice variant of *solute carrier organic anion transporter family member 1B3 (SLCO1B3)*. The other well-documented splice variant of *SLCO1B3* is liver-type organic anion transporting polypeptide 1B3 (Lt-OATP1B3), which is exclusively expressed in the human liver and mediates the hepatic uptake of various clinical drugs and endogenous compounds [3]. Compared to Lt-OATP1B3, *Ct-OATP1B3* mRNA has a different transcription start site and a shortened translation product [4, 5]. Recently, investigations have shown that both Ct-OATP1B3 mRNA and protein can be detected in several cancer tissues, including colorectal cancer, pancreatic cancer, and non-small cell lung cancer, but not in any normal tissues. Therefore, Ct-OATP1B3 is presumed to be an intriguing cancer-associated molecule that can be used in the development of cancer biomarker or therapeutic target [6]. However, its biological function in cancer is still unclear. As a variant of Lt-OATP1B3, Ct-OAPT1B3 is initially expected to have a transporter activity, but the results of several transport studies are inconsistent [5, 7, 8]. Therefore, an in-depth understanding of the function and underlying mechanisms of Ct-OATP1B3 in cancer are urgently needed.

Altered energy metabolism is a hallmark of cancer, in which the cells adapt their metabolism to fulfill the increased requirements for energy demands and biosynthetic intermediates. Although glycolysis is often enhanced in cancer cells, mitochondrial oxidative phosphorylation (OXPHOS) still plays a major role in energy production in many cancer cells, especially highly invasive cells, therapy-resistant cells, and cancer stem cells [9, 10]. Previous studies have demonstrated that over 90% of adenosine triphosphate (ATP) synthesis comes from OXPHOS in HGSOC cells, and more invasive HGSOC cells display more elevated OXPHOS activity [11, 12]. Moreover, there is compelling evidence showing that HGSOC cells also use fatty acid beta-oxidation (FAO) as an important energy source, and the enhanced FAO activity also can significantly promote HGSOC metastasis [13, 14]. However, the molecular mechanisms by which HGSOC cells alter their energy metabolism and affect metastasis remain obscure.

In this study, we demonstrated that overexpressed Ct-OATP1B3 in HGSOC promoted metastasis in vitro and in vivo. Ct-OATP1B3 upregulated the expression of carnitine palmitoyltransferase 1A (CPT1A) and NADH: Ubiquinone Oxidoreductase Subunit A2 (NDUFA2) by directly interacting with insulin-like growth factor 2 mRNA-binding protein 2 (IGF2BP2). Consequently, mitochondrial FAO and OXPHOS activities, as well as ATP production were increased, thus leading to cellular lamellipodia formation and HGSOC cell migration and invasion. These results have uncovered a novel mechanism contributing to the HGSOC metastasis, and provide evidence for Ct-OATP1B3 as a potential prognostic marker and therapeutic target for HGSOC patients.

## Results

### Ct-OATP1B3 is overexpressed in HGSOC and associated with progression and poor prognosis

**Fig. 1:**
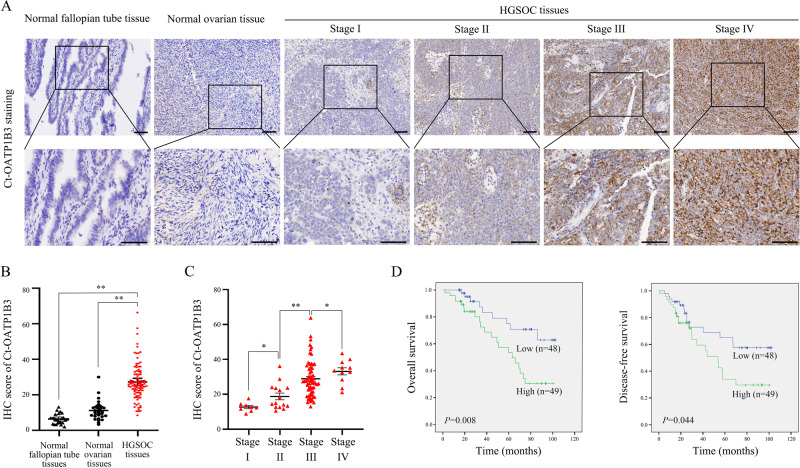
Increased Ct-OATP1B3 expression in HGSOC tissues is correlated with advanced tumor stage and poor clinical outcomes. **A** Representative images of Ct-OATP1B3 staining in normal fallopian tube samples, normal ovarian epithelial samples, and different FIGO-staged HGSOC tissues. Scale bar, 100 μm. **B** Quantification of Ct-OATP1B3 staining in 97 HGSOC and normal tissues. **C** Quantification of Ct-OATP1B3 staining in different FIGO-staged HGSOC tissues. **D** Kaplan–Meier curves for OS and DFS of HGSOC patients with low and high expression of Ct-OATP1B3. *P < 0.05, **P < 0.01.

The expression level of Ct-OATP1B3 in 97 HGSOC tissues, 29 normal ovarian samples, as well as 25 normal fallopian tube samples was firstly determined by tissue microarray analysis. The clinicopathological features of HGSOC patients are summarized in Table S1. Immunohistochemical (IHC) analysis showed that Ct-OATP1B3 was significantly highly expressed in HGSOC tissues compared with normal ovarian and fallopian tube samples (Fig. 1A and B). We next analyzed the relationship between Ct-OATP1B3 expression and clinicopathological parameters in HGSOC patients, and found that Ct-OATP1B3 overexpression was closely correlated with the Federation of Gynecologists and Obstetricians (FIGO) stage (*P* < 0.01), but was not significantly correlated with other parameters (Fig. 1A and C, Supplementary Table S2). Furthermore, Kaplan–Meier survival curves revealed that patients with a high level of Ct-OATP1B3 exhibited significantly shorter overall survival (OS) and disease-free survival (DFS) than those with a low level (Fig. 1D). Cox regression analysis further revealed that high Ct-OATP1B3 expression was an independent predictor of poor survival in patients with HGSOC (Supplementary Table S3).

As an alternative splicing product of *SLCO1B3* gene, *Ct-OATP1B3* mRNA has a unique first exon which locates at the second intron of *SLCO1B3*. Therefore, the predicted translation product of *Ct-OATP1B3* mRNA lack the N-terminal 28 amino acid of Lt-OATP1B3 [4–6]. Since the epitope of antibody against Ct-OATP1B3 used in this study is localized at the C-terminal of Ct-OATP1B3, therefore this antibody can also be applied to detect Lt-OATP1B3. To exclude the interference of Lt-OATP1B3, we detected the mRNA levels of both *Ct-OATP1B3* and *Lt-OATP1B3* in HGSOC tissues, normal ovarian samples, as well as normal fallopian tube tissues. Consistent with the data from other types of cancer, we found that the level of *Lt-OATP1B3* mRNA was very low in all the tissues examined, with no significant difference among them. However, the level of *Ct-OATP1B3* mRNA was several thousand-fold higher than that of *Lt-OATP1B3* in HGSOC tissues, despite it also remained at a low level in normal ovarian and fallopian tube tissues [4, 5, 7] (Fig. S1A). Therefore, Lt-OATP1B3 only has negligible impact on our observations.

Then, we compared the expression of Ct-OATP1B3 between HGSOC cell lines (OVCAR3, SKOV3, OVCAR4, CAOV3) and normal ovarian surface epithelium HOSE. Our results also suggested that both Ct-OATP1B3 mRNA and protein were hardly detectable in HOSE cells, but significantly increased in all four HGSOC cell lines. Among them, OVCAR3 showed the highest level of Ct-OATP1B3 expression, while CAOV3 showed the lowest. As for Lt-OATP1B3, its expression could not be detected in all the cell lines tested, but significantly higher in liver tissue (Fig. S1B and C). Taken together, these data suggest that the expression of Ct-OATP1B3 is significantly upregulated in HGSOC, and positively correlated with poor prognosis in HGSOC patients.

### Ct-OATP1B3 plays a key role in HGSOC cell migration and invasion

**Fig. 2:**
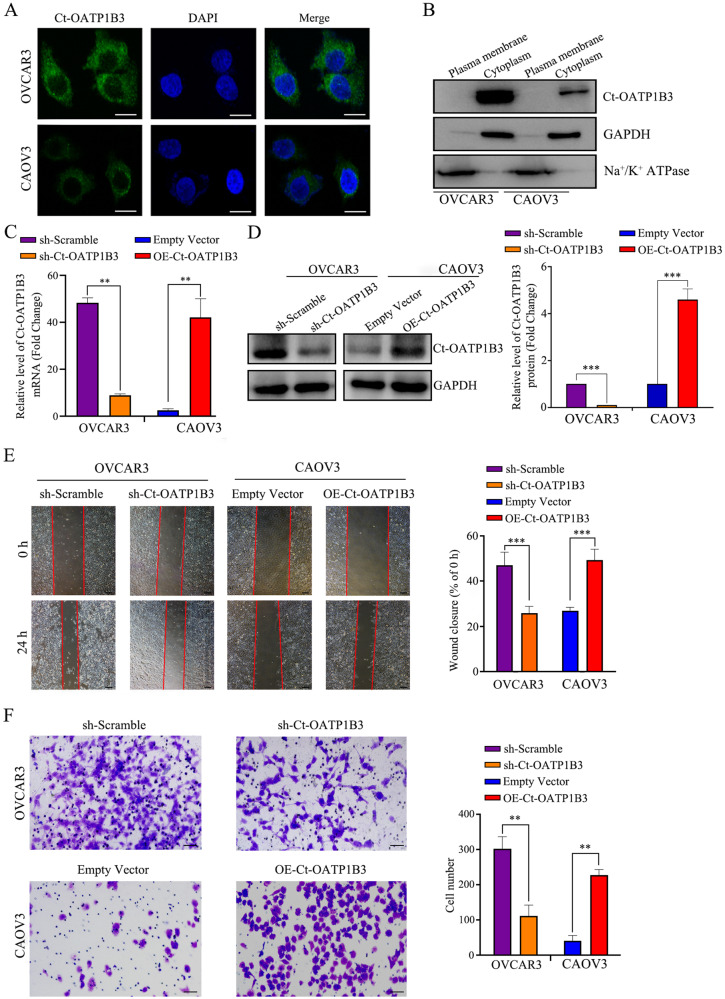
Ct-OATP1B3 promotes the invasion and migration of HGSOC cells. **A**, **B** Subcellular localization of Ct-OATP1B3 in HGSOC cells. Scale bar, 25 μm. **C**, **D** Ct-OATP1B3 expression in HGSOC cells after Ct-OATP1B3 overexpression or knockdown. **E**, **F** Ct-OATP1B3 promotes the migration (**E**, scale bar, 500 μm) and invasion (**F**, scale bar, 200 μm) of HGSOC cells. **P < 0.01, ***P < 0.001.

To explore the biological function of Ct-OATP1B3 in HGSOC cells, we first observed its subcellular location in HGSOC cells. Using immunofluorescence staining and western blot, we confirmed that Ct-OATP1B3 protein was localized mainly in the cytoplasm, but not on the plasma membrane of HGSOC cells (Fig. 2A and B). Therefore, we speculated that Ct-OATP1B3 might not act as a transporter. As expected, using cholecystokinin-8 (CCK-8) and paclitaxel as substrates, we found that Ct-OATP1B3 had no, or at best a very weak transport activity (Fig. S1D and E).

Then, we observed the influence of Ct-OATP1B3 on the proliferation, apoptosis, adhesion, migration, and invasion of HGSOC cells. Our results showed that Ct-OATP1B3 overexpression significantly accelerated the migration speed and invasion ability of CAOV3 cells, while OVCAR3 cells exhibited repressed migration and invasion capacities after Ct-OATP1B3 knockdown (Fig. 2C–F). Besides, Ct-OATP1B3 also exert a relatively weaker but significant pro-proliferation activity (Supplementary Fig. S1F). However, no effects of Ct-OATP1B3 were found on the apoptosis and adhesion of HGSOC cells (Supplementary Fig. S1G and H). These results indicate that Ct-OATP1B3 mainly contributes to the migration and invasion of HGSOC cells.

### Ct-OATP1B3 promotes HGSOC cell migration and invasion through interacting with IGF2BP2

**Fig. 3:**
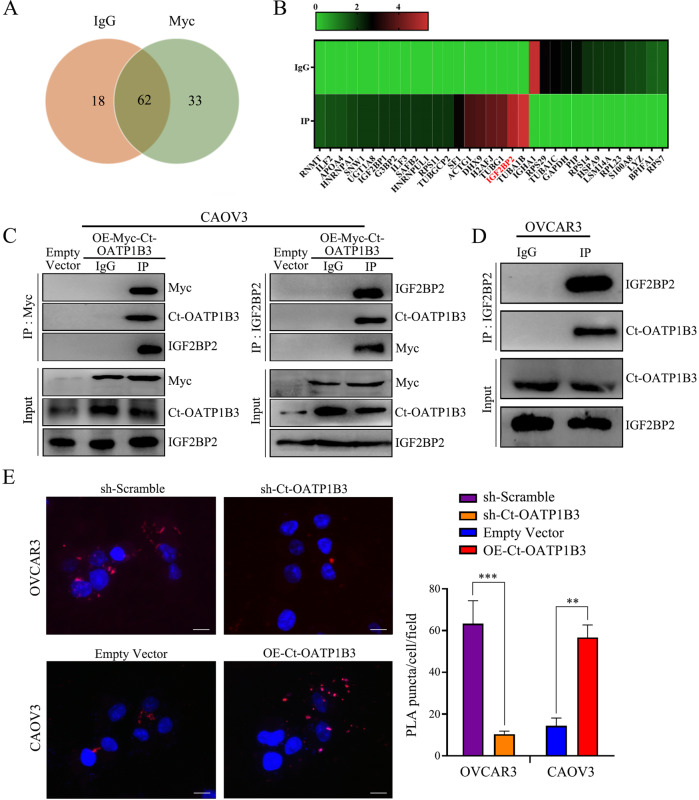
Ct-OATP1B3 directly interacts with IGF2BP2 in HGSOC cells. **A**, **B** Venn diagrams and Heatmap showing the candidate proteins interacting with Ct-OATP1B3. **C** Reciprocal Co-IP analysis showing the Ct-OATP1B3-IGF2BP2 complex in myc-Ct-OATP1B3 overexpressing CAOV3 cells. **D** Co-IP analysis showing the endogenous Ct-OATP1B3-IGF2BP2 interaction in OVCAR3 cells. **E** In situ PLA reveals the direct physical interaction between Ct-OATP1B3 and IGF2BP2 in HGSOC cells. Scale bar, 20 μm. **P < 0.01, ***P < 0.001.

To explore the mechanisms underlying the role of Ct-OATP1B3 in HGSOC cell migration and invasion, we applied affinity purification and mass spectrometry analysis to identify Ct-OATP1B3 interacting proteins. Thirty-three proteins were shown to be potential interacting partners of Ct-OATP1B3, and IGF2BP2 was shown to be one of the top candidates (Fig. 3A and B).

To confirm the interaction between Ct-OATP1B3 and IGF2BP2 in HGSOC cells, we first performed Co-immunoprecipitation (Co-IP) assay. In myc-Ct-OATP1B3 overexpressing CAOV3 cells, IGF2BP2 could be co-IPed with anti-myc antibody, and reciprocally, myc-Ct-OATP1B3 could also be co-IPed with anti-IGF2BP2 antibody (Fig. 3C). In OVCAR3 cells, endogenous Ct-OATP1B3 could also interact with endogenous IGF2BP2 (Fig. 3D). To further validate the close-range interaction between Ct-OATP1B3 and IGF2BP2, we applied in situ proximity ligation assay (PLA). A positive PLA result relies on the distance between the two proteins is <40 nm, which reflects true protein-protein interaction [15]. As shown in Fig. 3E, more Ct-OATP1B3-IGF2BP2 interactions occurred in Ct-OATP1B3 overexpressing CAOV3 cells (*P* < 0.01), while Ct-OATP1B3 knockdown significantly reduced Ct-OATP1B3-IGF2BP2 interactions in OVCAR3 cells (*P* < 0.001). Together, these results support a direct interaction between Ct-OATP1B3 and IGF2BP2 in HGSOC cells.

**Fig. 4:**
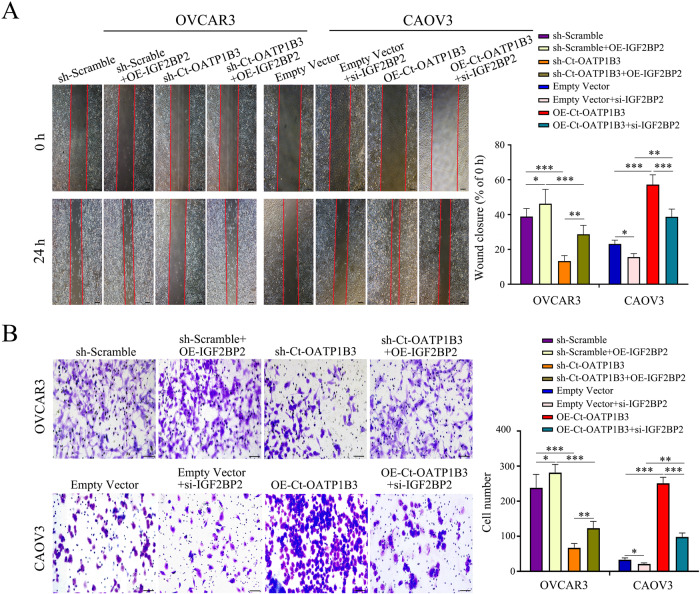
Ct-OATP1B3 promotes migration and invasion of HGSOC cells through interacting with IGF2BP2. **A** Ct-OATP1B3 promotes migration of HGSOC cells in an IGF2BP2-dependent manner. Scale bar, 500 μm. **B** Ct-OATP1B3 promotes invasion of HGSOC cells in an IGF2BP2-dependent manner. Scale bar, 200 μm. *P < 0.05, **P < 0.01, ***P < 0.001.

Next, we explored whether Ct-OATP1B3 promoted migration and invasion of HGSOC cells in an IGF2BP2-dependent manner. As expected, our rescue experiments revealed that IGF2BP2 could significantly reverse the effect of Ct-OATP1B3 on the migration and invasion capacities of HGSOC cells (Fig. 4A and B). As an RNA-binding protein, IGF2BP2 (also known as IMP2) belongs to IGF2BPs family, which can bind to various transcripts and regulate their subcellular stability, localization, and translation. Recent evidence has indicated that IGF2BP2 contributes to the progression of several cancers, including liver cancer, breast cancer, ovarian cancer, *etc* [16]. Here, we also found IGF2BP2 expression was significantly upregulated in HGSOC tissues compared with normal ovarian samples. A similar result was obtained from Gene Expression Profiling Interactive Analysis (GEPIA) database (http://gepia.cancer-pku.cn/) analysis (Fig. S2A–C). Importantly, we found a positive association between the expression levels of Ct-OATP1B3 and IGF2BP2, supporting a close relationship between Ct-OATP1B3 and IGF2BP2 in HGSOC progression (Fig. S2D). Collectively, these data support that Ct-OATP1B3 promotes the HGSOC metastasis through interacting with IGF2BP2.

### Ct-OATP1B3 facilitates IGF2BP2 binding to CPT1A and NDUFA2

**Fig. 5:**
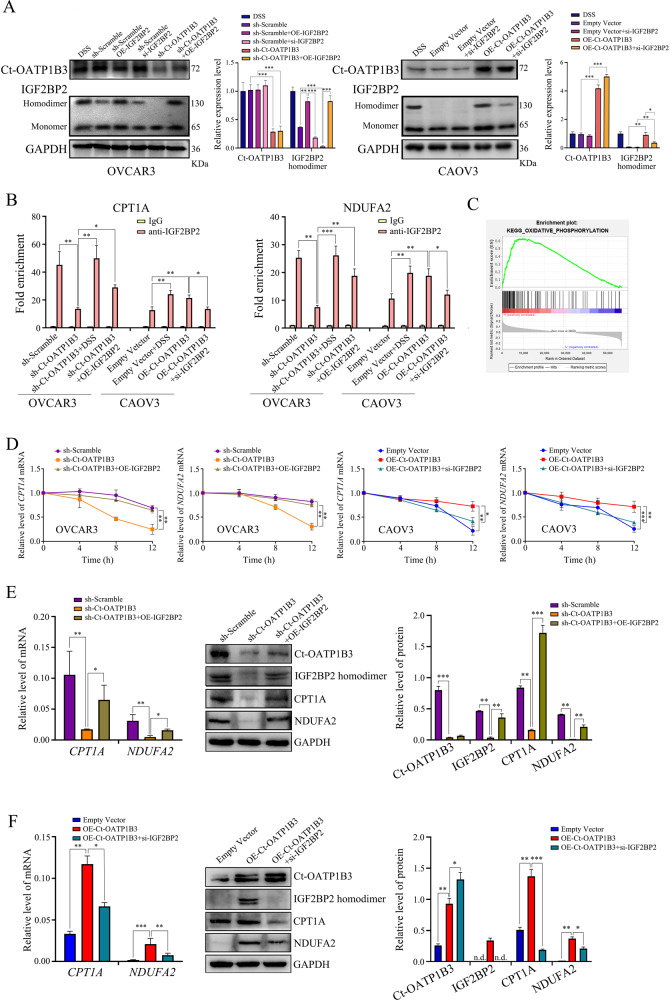
Ct-OATP1B3 facilitates IGF2BP2 binding to its mRNA targets. **A** Ct-OATP1B3 promotes the formation of IGF2BP2 homodimer in HGSOC cells. **B** RIP assay shows Ct-OATP1B3 enhances the binding of IGF2BP2 to its mRNA targets *CTP1A* and *NDUFA2*. **C** GSEA analysis demonstrates that increased expression of Ct-OATP1B3 in HGSOC tissues is related to the mitochondrial OXPHOS pathway. **D** Ct-OATP1B3 enhances *CPT1A* and *NDUFA2* mRNA stabilities after actinomycin D treatment. **E**, **F** Ct-OATP1B3 enhances CPT1A and NDUFA2 expression in OVCAR3 (**E**) and CAOV3 (**F**) cells. *P < 0.05, **P < 0.01, ***P < 0.001.

Since our data indicated that Ct-OATP1B3 directly interacted with IGF2BP2, and Ct-OATP1B3 promoted migration and invasion of HGSOC cells in an IGF2BP2-mediated manner, we, therefore, investigated whether Ct-OATP1B3 could modulate the function of IGF2BP2, attempting to elucidate the mechanism underlying this phenomenon. All members of the IGF2BP protein family have four C-terminal heterogeneous nuclear ribonucleoprotein (hnRNP)-K homology (KH) domains. Previous studies have suggested that the IGF2BP1, another member of IGF2BPs family, can form homodimer through KH3 and KH4 domains, and the dimerization of IGF2BP1 might allow it to interact more stably with the target mRNAs [17, 18]. Accordingly, we hypothesized that IGF2BP2 also could form homodimer, and Ct-OATP1B3 might regulate the IGF2BP2 dimer formation in HGSOC cells. To study this, we first assessed the association of two forms of IGF2BP2 protein tagged with either HA or Myc epitope by Co-IP after their coexpression in HEK293T cells. Our results demonstrated the association of HA-IGF2BP2 with Myc-IGF2BP2 in the cells, and more importantly, Ct-OATP1B3 could enhance the interaction between HA-IGF2BP2 and Myc-IGF2BP2 (Fig. S3A). Moreover, we found that disuccinimidyl suberate (DSS), a chemical cross-linking reagent that is commonly used for protein homodimerization [19], also significantly facilitated the formation of GST-tagged IGF2BP2 homodimers in HEK293T cells (Fig. S3B). Based on these results, we decided to detect the influence of Ct-OATP1B3 on the formation of endogenous IGF2BP2 homodimer in HGSOC cells. Our results showed that in OVCAR3 cells, Ct-OATP1B3 knockdown dramatically reduced the formation of IGF2BP2 homodimer, which could be rescued by IGF2BP2 overexpression. While in CAOV3 cells, Ct-OATP1B3 overexpression markedly increased the IGF2BP2 homodimer formation, which could be abolished by IGF2BP2 knockdown (Fig. 5A).

IGF2BP2 has some well-described mRNA targets, particularly those associated with mitochondrial metabolism, including *CPT1A* and *NDUFA2* [20, 21]. Using RIP assay, we found that Ct-OATP1B3 strongly promoted IGF2BP2 binding to *CPT1A* and *NDUFA2* mRNAs, indicating that Ct-OATP1B3 promotes the mRNA-binding activity of IGF2BP2 via enhancing the IGF2BP2 homodimers formation (Fig. 5B). Interestingly, using Gene Set Enrichment Analysis (GSEA), we also found that increased expression of Ct-OATP1B3 in HGSOC tissues were significantly related to mitochondrial metabolism (Fig. 5C).

Given that IGF2BP2 was reported to stabilize mRNA through direct binding [16], we wondered whether Ct-OATP1B3 could regulate the mRNA stability and expression of *CPT1A* and *NDUFA2* through interacting with IGF2BP2. After being treated with actinomycin D, an inhibitor of transcription, we found that Ct-OATP1B3 significantly increased the stability of *CPT1A* and *NDUFA2* mRNAs, leading to enhanced expression of CPT1A and NDUFA2 (Fig. 5D–F). Thus, these data indicate that Ct-OATP1B3 enhances the binding of IGF2BP2 with *CPT1A* and *NDUFA2* mRNAs, and promotes the expression of these two transcripts, giving us a hint that Ct-OATP1B3 might regulate the mitochondrial metabolism in HGSOC cells.

### Ct-OATP1B3 promotes FAO and OXPHOS activities and lamellipodia formation in HGSOC cells

**Fig. 6:**
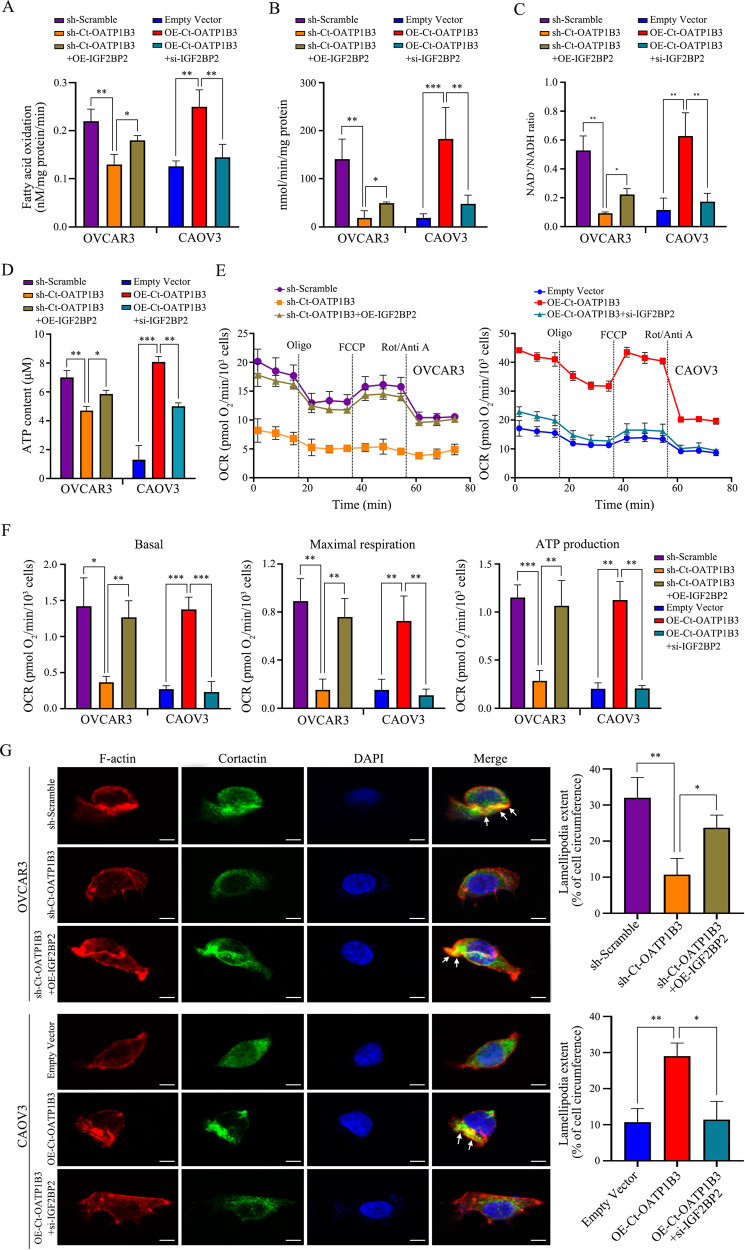
Ct-OATP1B3 enhances the mitochondrial FAO and OXPHOS activities and promotes lamellipodia formation in HGSOC cells. **A** Ct-OATP1B3 increases FAO oxidation rate in HGSOC cells. **B**–**D** Ct-OATP1B3 increases mitochondrial complex I activity (**B**), NAD^+^/NADP ratio (**C**), intracellular ATP level (**D**) in HGSOC cells. **E**, **F** Seahorse assays demonstrate that Ct-OATP1B3 promotes OCR in HGSOC cells. **G** Ct-OATP1B3 promotes the formation of lamellipodia in HGSOC cells. Lamellipodia extents at cell edges are quantified as a percentage of the cell circumference on 100 randomly selected cells in each group. Arrows indicate sites of lamellipodia extension. Scale bar, 10 μm. *P < 0.05, **P < 0.01, ***P < 0.001.

CPT1A is a critical rate-limiting enzyme of FAO, and its increased expression can elevate the FAO rate [22]. To test whether Ct-OATP1B3 could induce FAO activity in HGSOC cells, relative FAO rate was accessed using [^3^H]-labeled palmitate as a tracer. As shown in Fig. 6A, Ct-OATP1B3 knockdown significantly reduced FAO rate (*P* < 0.01) in OVCAR3 cells, which could be rescued by IGF2BP2 overexpression. Similarly, Ct-OATP1B3 overexpression in CAOV3 cells resulted in a markedly increase in FAO activity (*P* < 0.01), but IGF2BP2 knockdown reversed this increase (Fig. 6A). These results indicate HGSOC cells’ dependence on Ct-OATP1B3 for FAO activation.

NDUFA2 is an accessory subunit of NADH:ubiquinone oxidoreductase (complex I), the first and largest complex of the mitochondrial respiratory chain. It plays a critical role in the assembly of complex I, which further affect the mitochondrial OXPHOS activity [23]. Since Ct-OATP1B3 was also shown to increase the expression of NDUFA2, we explored the influence of Ct-OATP1B3 on mitochondrial OXPHOS in HGSOC cells. We first found that Ct-OATP1B3 significantly increased complex I activity, NAD^+^/NADP ratio, as well as intracellular ATP level (Fig. 6B–D). Then, oxygen consumption rate (OCR) was further measured by Seahorse analysis, and the results demonstrated that Ct-OATP1B3 significantly increased the basal, maximal, and ATP-linked OCR in HGSOC cells, which could be rescued by IGF2BP2 silence (Fig. 6E and F). These data suggest that Ct-OATP1B3 promotes the OXPHOS activity and ATP production in HGSOC cells.

As previously reported, a substantial amount of ATP is necessary for reorganizing and reassembling polymeric actin (F-actin) filaments, which are the driving force for the formation of lamellipodia, a sheet-like protrusion at the leading edge of tumor cells, which is a key structure for cancer cell migration and invasion [24–26]. Since our findings established that Ct-OATP1B3 enhanced mitochondrial FAO and OXPHOS activities and elevated ATP production, we next investigated whether Ct-OATP1B3 could facilitate the formation of lamellipodia. Using phalloidin (a probe for F-actin) and cortactin (a marker for lamellipodia) staining [27], we found that Ct-OATP1B3 markedly promoted F-actin cytoskeletal assembly and increased the extent of lamellipodia, which could be rescued by IGF2BP2 knockdown (Fig. 6G). These data suggest that Ct-OATP1B3 promotes FAO and OXPHOS activities, ATP production and lamellipodia formation in HGSOC cells, which can explain its impact on HGSOC cell migration and invasion.

### Ct-OATP1B3 promotes HGSOC metastasis in vivo

**Fig. 7:**
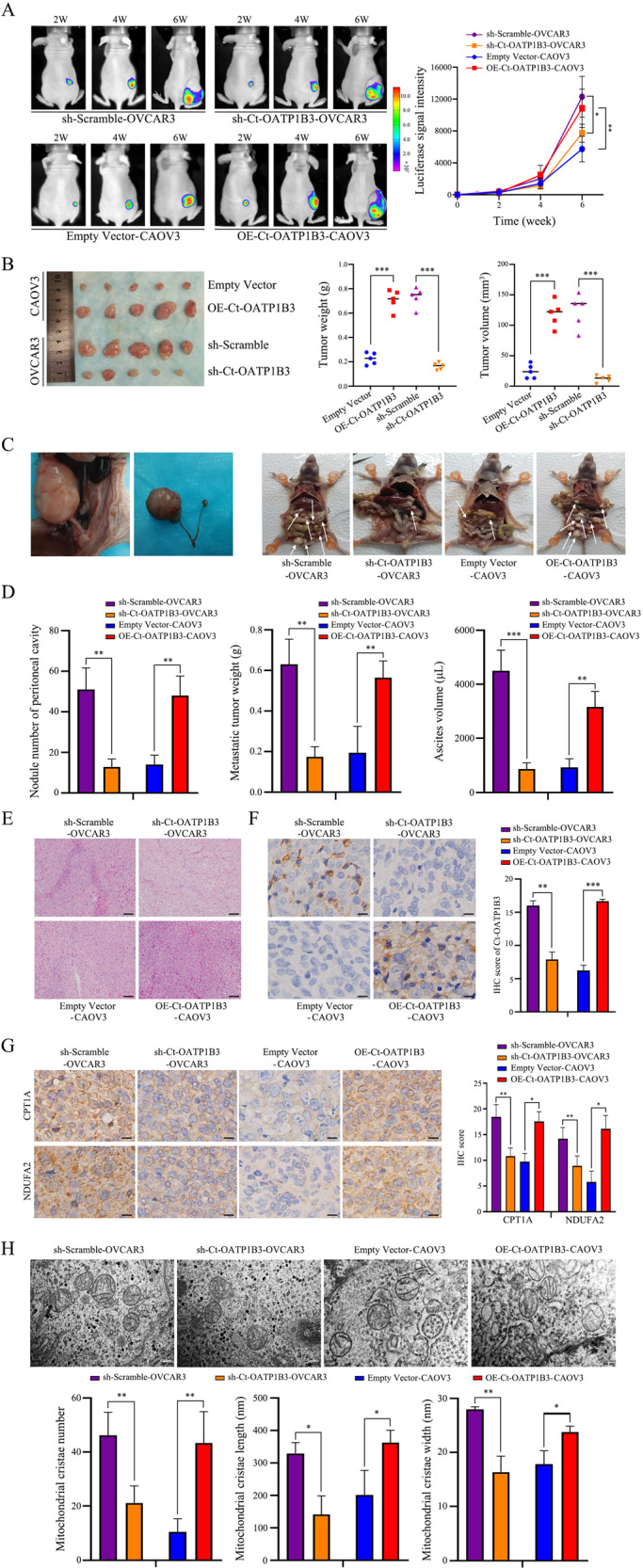
Ct-OATP1B3 promotes HGSOC metastasis in vivo. **A** Representative bioluminescence images of orthotopic mouse models. **B** Tumor growth in different groups. **C** Representative pictures of peritoneal metastasis in orthotopic mouse models. Ct-OATP1B3 increases the peritoneal metastasis burden. Arrows indicate peritoneal metastases. **D** Ct-OATP1B3 increases the metastatic nodule number, tumor weight, and ascites volume in orthotopic mouse models. **E** Representative H&E staining of the orthotopic tumors in different groups. Scale bar, 100 μm. **F** Representative images of the Ct-OATP1B3 staining in different groups of orthotopic tumors. Scale bar, 25 μm. **G** Representative images of the CPT1A and NDUFA2 staining in different groups of orthotopic tumors. Scale bar, 25 μm. **H** Ultrastructural analysis of mitochondrial cristae in different groups of orthotopic tumors. Scale bar, 200 nm. *P < 0.05, **P < 0.01, ***P < 0.001.

To further assess the impact of Ct-OATP1B3 on HGSOC metastasis in vivo, we established orthotopic xenograft models of HGSOC. Ct-OATP1B3-knockdown or -overexpression HGSOC cells were orthotopically transplanted into the right ovaries of nude mice, and the tumor growth was monitored by an in vivo imaging system (IVIS). Ct-OATP1B3-knockdown group (sh-Ct-OATP1B3-OVCAR3) exhibited reduced tumor volume and tumor weight, whereas Ct-OATP1B3 overexpression group (OE-Ct-OATP1B3-CAOV3) demonstrated a significant increase of tumor volume and tumor weight, when compared to their respective controls (Fig. 7A and B). Consistent with this, the metastatic nodule number, metastatic tumor weight, and ascites volume in mice bearing sh-Ct-OATP1B3-OVCAR3 cells significantly decreased, whereas that in mice bearing OE-Ct-OATP1B3-CAOV3 cells obviously increased, when compared to those of the respective control (Fig. 7C and D). In conclusion, these findings indicate that Ct-OATP1B3 contributes to HGSOC metastasis in vivo.

To further verify the effect of Ct-OATP1B3 on HGSOC metastasis in vivo is related to the modulation of FAO and OXPHOS activities, we analyzed the expression of CPT1A and NDUFA2 in orthotopic tumors. As expected, CTP1A and NDUFA2 were significantly downregulated in Ct-OATP1B3 knockdown group, and markedly upregulated in Ct-OATP1B3 overexpression group (Fig. 7E–G). Furthermore, we also observed the Ct-OATP1B3-induced mitochondrial cristae alterations. Previous studies have reported that the number and morphology of cristae reflect the response of the mitochondria to the cellular energy demands. Increased cristae number and length, and decreased cristae width not only reflect the enhanced OXPHOS, but also represent the increased activity of FAO [28, 29]. Ultrastructural analyses revealed that Ct-OATP1B3 significantly increased the number and length of cristae, while reduced the width of cristae in orthotopic tumors (Fig. 7H). Collectively, these data suggest the facilitation effect of Ct-OATP1B3 on HGSOC metastasis is related to the upregulation of mitochondrial FAO and OXPHOS activities in vivo.

## Discussion

HGSOC is the most common and deadly subtype of ovarian cancer. Recent research has demonstrated that both fallopian tube and ovarian surface epithelium are cells-of-origin for HGSOC [30]. Despite extensive research efforts, very few prognostic markers and therapeutic targets of HGSOC have been successfully implemented into clinical practice so far [31].

In the present study, elevated expression of Ct-OATP1B3 was found in HGSOC tissues compared with normal fallopian tubes and normal ovarian tissues, which was clearly associated with advanced tumor stage and poor patient outcomes. Moreover, a high level of Ct-OATP1B3 is an independent poor prognostic factor in HGSOC patients. Using in vitro culture studies, we also found that Ct-OATP1B3 was highly expressed in HGSOC cell lines, but not in HOSE cells. This conclusion is consistent with some previous research showing that Ct-OATP1B3 may represent a novel indicator for the progression of some types of cancer. For example, *Ct-OATP1B3* mRNA has been proved to be present in serum and cancerous tissues of colorectal cancer patients, and can be used as a diagnosis biomarker. In non-small cell lung cancer tissues, Ct-OATP1B3 protein is also overexpressed and acts as a tumor-promoting factor [32–34]. However, the present research has limitation in sample size, and it is a single-center study. Moreover, SKOV3 is considered not the appropriate model for HGSOC recently [35]. Therefore, further multi-center studies with a larger sample size, as well as more suitable HGSOC cell lines are needed to provide more convincing evidence for the clinical significance of Ct-OATP1B3 in HGSOC.

Despite the important role of Ct-OATP1B3 in critical processes for tumor progression, the biological function and underlying mechanisms remain poorly understood. In this study, we observed that Ct-OATP1B3 has no transport activity, and may have additional roles other than a potential transporter-like function. Subsequently, we found that Ct-OATP1B3 promoted the migration and invasion of HGSOC cells in vitro, mechanically through interacting with IGF2BP2. IGF2BP2 has been shown to be overexpressed in many cancers and associated with poor patient survival. For example, *IGF2BP2* gene is a relatively common event in comparison to the amplification of the other IGF2BPs family members, *IGF2BP1* and *IGF2BP3*, occurring in ~15–27% of ovarian cancers [16, 36]. Here, we also found IGF2BP2 was upregulated in HGSOC tissues compared with normal ovarian tissues. What’s more, its expression level was positively associated with that of Ct-OATP1B3 in HGSOC tissues. Recently, a growing number of studies has shown that upregulated IGF2BP2 in cancer cells play a critical role in cell migration, invasion, and metabolic alteration. For example, high expression of IGF2BP2 in hepatocellular carcinoma can promote cell migration and invasion abilities by activating the Wnt/β-catenin pathway. Moreover, as a mRNA-binding protein, IGF2BP2 also regulates aerobic glycolysis by directly binding to and stabilizing the mRNAs of glycolysis-related genes, such as *Glucose transporter 1 (GLUT1)* [37, 38].

Then, we observed that Ct-OATP1B3 could regulate the activity of IGF2BP2 through facilitating its homodimerization. Therefore, we were not surprised to find that the expression levels of two IGF2BP2 target mRNAs, *CPT1A* and *NDUFA2*, were elevated following Ct-OATP1B3 overexpression. As the key players in the processes of mitochondrial FAO and OXPHOS, the elevated CPT1A and NDUFA2 significantly promoted FAO and OXPHOS activities in HGSOC cells. It has been demonstrated that, dysregulation of several genes, such as fatty acid-binding protein 4, collagen XI alpha 1, and paired box 2, is closely related to the enhancement of FAO activity in HGSOC cells [13, 39, 40]. While increased mitochondrial DNA copy number and enhanced cellular pyruvate uptake are proved to contribute to OXPHOS upregulation [11, 41]. In this study, we found Ct-OATP1B3 was also a modulator of FAO and OXPHOS pathways. Given that mitochondrial FAO and OXPHOS have been considered as druggable targets for cancer treatment [42, 43], Ct-OATP1B3 might be a new choice that can be used to interfere with these pathways with high specificity because of its predominant or exclusive expression in many kinds of cancer cells.

It is well known that elevated FAO and/or OXPHOS activities lead to increased ATP production, and increased ATP concentration promotes cellular lamellipodia formation. As one of the formations of extensions of the cell membrane, lamellipodia are located at the leading edge of the migrating cells with the ability to sense the surrounding environment, drive and guide cell locomotion. Therefore, the formation of lamellipodia is the first step in the processes of tumor cell migration and invasion [24–26, 44]. Increased mitochondrial function plays a critical role in promoting lamellipodia formation in many kinds of cancer cells, such as breast cancer, ovarian cancer, and liver cancer, which leads to cancer metastasis [45–47]. In this study, we found Ct-OATP1B3-induced mitochondrial FAO and OXPHOS enhancements facilitated the lamellipodia formation, which provides a reasonable explanation for the metastasis-promoting role of Ct-OATP1B3 in HGSOC cells.

**Fig. 8:**
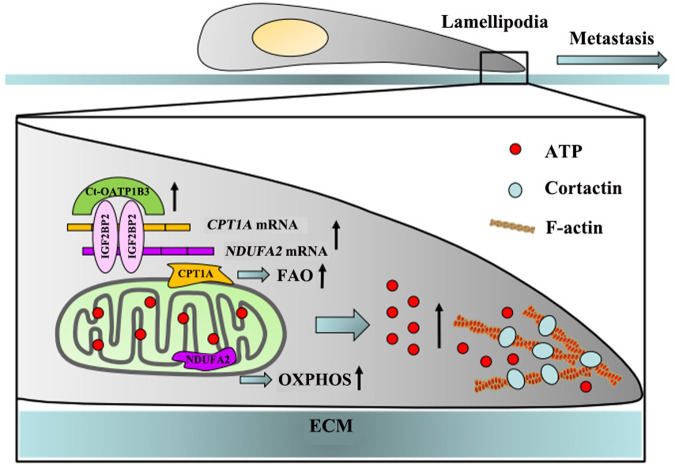
Schematic of the function and mechanism of Ct-OATP1B3 in HGSOC metastasis. Ct-OATP1B3 directly interacts with IGF2BP2 and increases the expression of CPT1A and NDUFA2, which enhances the FAO and OXPHOS activities and ATP production, thus leading to the lamellipodia formation and HGSOC cell migration and invasion.

In conclusion, our studies reveal that Ct-OATP1B3 is critical for HGSOC metastasis. Ct-OATP1B3 interacts with IGF2BP2 and increases the expression of CPT1A and NDUFA2, which enhances the FAO and OXPHOS activities and ATP production, thus leading to the lamellipodia formation and HGSOC cell migration and invasion (Fig. 8). Our findings support that Ct-OATP1B3 can be utilized as a promising diagnostic marker and therapeutic target for HGSOC patients.

## Materials and methods

### Patients and tissue samples

HGSOC tissues, normal ovarian tissues as well as normal fallopian tube samples were collected by the Tissue Bank of the Obstetrics and Gynecology Hospital (Fudan University in Shanghai, China). The patients’ characteristics are summarized in Table S1. Written informed consent was obtained from all participants. The study protocol was approved by the institutional review board of the Obstetrics and Gynecology Hospital of Fudan University (Approval Number: 2018-24). The procedures of tissue microarray (TMA) construction were described previously [48].

### Cell culture and treatment

The human ovarian surface epithelium (HOSE), HGSOC cell lines, and HEK293T cells were obtained from the Cell Bank of Type Culture Collection of the Chinese Academy of Sciences. OVCAR3, OVCAR4, and HOSE cells were cultured in 1640 medium, CAOV3 and HEK293T cells were cultured in DMEM medium, SKOV3 cells were cultured in McCoy’s 5A medium supplemented with 10% fetal bovine serum (FBS, Gibco, USA). Cell lines were regularly tested for mycoplasma, and were authenticated at source by STR profiling, morphology (ATCC), and DNA profiling (ECACC).

### Quantitative real-time PCR

Quantitative real-time PCR was performed using TB Green Premix Ex Taq II (TaKaRa, Japan). The primer sequences used in this study are shown in Table S4. GAPDH served as an internal control.

### Immunohistochemistry (IHC)

The de-paraffinized sections were incubated with 20% goat serum for 30 min and were incubated with primary antibodies against Ct-OATP1B3 (1:200, Abcam, USA), IGF2BP2 (1:100, Santa Cruz, USA), CPT1A (1:1000, Abcam, USA), and NDUFA2 (1:100, Abcam, USA) at 4 °C overnight followed by the secondary antibody for 1 h at 37 °C. The calculation method of IHC score was performed as described previously [49]. Ct-OATP1B3 expression level was analyzed by classifying IHC score as low (based on the IHC score lower than the median value) and as high (based on an IHC score higher than the median value).

### Immunofluorescent (IF) assay

To observe Ct-OATP1B3 subcellular localization, cells were incubated with primary antibodies against Ct-OATP1B3 (1:100, Santa Cruz, USA) at 4 °C overnight, then incubated with secondary antibody at 37 °C for 1 h and co-stained with DAPI for 5 min at room temperature. To observe cellular lamellipodia, cells were incubated with anti-cortactin antibody (1:100, Bioss, China) for overnight at 4 °C, then treated with 100 nM TRITC Phalloidin (Solarbio, China) for 30 min. Cell micrographs were obtained using a Zeiss Axioplan 2 imaging microscope (Zeiss AG, Oberkochen, Germany).

### Western blot analysis

The protein extracts were separated by 8% SDS-PAGE and transferred onto polyvinylidene difluoride (PVDF) membranes (Bio-Rad, USA). The membranes were incubated with primary antibodies as follows: Ct-OATP1B3 (1:1000, Abcam, USA), Tubulin (1:1000, Beyotime, China), Na^+^/k^+^ ATPase (1:1000, Beyotime, China), GAPDH (1:5000, Bioss, China), Myc (1:1000, Cell Signaling Technology, USA), IGF2BP2 (1:1000, Cell Signaling Technology, USA), CPT1A (1:1000, Abcam, USA), NDUFA2 (1:1000, Abcam, USA). After incubation with secondary antibodies, immunoreactive bands were detected by ECL^TM^ Prime (Millipore, USA) and a LAS-3000 imager (Bio-Rad, USA).

### DNA constructs

Full-length cDNA encoding human *Ct-OATP1B3* and *IGF2BP2* were amplified by PCR and cloned into the cs-U0996-Lv217-01 lentiviral vector system. A Ct-OATP1B3-specific shRNA (sequence 5′-GGAATTGGTTGTCTCCTTA-3′) was inserted into the cs-HSH099030-LvRU6Rlp-01 lentiviral vector system. The sequences of *IGF2BP2* siRNA are as follows: si-IGF2BP2-1, CCCGCAUCAUCACUCUUAUTT; si-IGF2BP2-2: AUAAGAGUGAUGAUGCGG. These plasmids or siRNA were transfected into cells using Lipofectamine^TM^ 3000 reagent (Invitrogen, USA).

### Cell migration and invasion assays

Cell migration was measured using a wound-healing assay. Cells were seeded in 24-well plates and were cultured at 37 °C to reach a 90% confluence. Linear scratch wounds were created by 200 μL pipette tip. Then the plate was washed by PBS and the cells were cultured in serum-free media. After 24 h, the plates were observed for wound healing and the average migration distance of the cells were measured using the ImageJ software. For cell invasion assay, the upper chamber of transwell insert was pre-coated with Matrigel (Corning, USA). Cells were seeded in the upper chamber in a serum-free medium. After 48 h incubation, the cells on the lower side were fixed with 4% paraformaldehyde, stained with 0.5% crystal violet, and counted.

### Co-immunoprecipitation and LC-MS/MS analysis

Whole-cell lysate of myc-Ct-OATP1B3 overexpressing CAOV3 cells was prepared using RIPA P0013 lysis buffer (Beyotime, China) supplemented with protease and phosphatase inhibitors (Roche Diagnostics, Germany). After incubation on ice for 30 min and centrifugation (12,000 g at 4 °C) for 15 min, the supernatant was precleared with Protein A/G-Sepharose at 4 °C for 2 h. Myc-Ct-OATP1B3 interacting partners were co-immunoprecipitated with anti-Myc antibody covalently coupled to Protein A/G-Sepharose. The subsequent LC-MS/MS measurements were performed as previously described [50].

### In situ PLA

PLA was performed using DuoLink PLA kit (Sigma-Aldrich, USA) according to the manufacturer’s instructions. Briefly, cells were fixed using 4% paraformaldehyde followed by permeabilization with 0.5% Triton X-100. After treatment with DuoLink blocking buffer, cells were incubated with rabbit anti-Ct-OATP1B3 (1:50, Santa Cruz, USA) and mouse anti-IGF2BP2 (1:100, Santa Cruz, USA) primary antibodies, and oligonucleotide-labeled anti-mouse and anti-rabbit secondary antibodies (PLA probes). Subsequently, cells were treated with ligation-ligase solution and Amplification-Polymerase solution at 37 °C. To localize PLA signals, cells were co-stained using DAPI, and PLA signals (red puncta) were counted on confocal images.

### Chemical cross-linking and IGF2BP2 homodimer detection

Cells with or without transfection of various Ct-OATP1B3 or IG2BP2 constructs were harvested, and the cell suspension was incubated with 1.25 mM disuccinimidyl suberate (DSS). For the preparation of mock lysates, DMSO without DSS was added to the cells. Cross-linking was carried out for 30 min at 37 °C, followed by terminating of the cross-linking with Tris-buffered saline (TBS at 25 mM, pH 7.5) for 15 min. The monomer (MW 65 kDa) and homodimer (MW 130 kDa) of IGF2BP2 were analyzed by nondenaturing PAGE. Briefly, cells were lysed in Nondenaturing Lysis Buffer (Biohao, Wuhan, China). After centrifugation at 11,000 × *g* for 10 min, the supernatants were separated by electrophoresis on a 15% Tris/glycine polyacrylamide gel followed by transfer to PVDF membrane for Western blot analysis [51].

### RNA immunoprecipitation (RIP) assay

The RIP assay was performed using Magna RIP RNA-binding protein immunoprecipitation kit (Merck Millipore, Germany) according to the manufacturer’s instructions. Briefly, cells were collected and lysed in RIPA buffer with protease and RNase inhibitors. Cell lysates were incubated with magnetic beads conjugated with IGF2BP2 antibodies or IgG (Millipore) at 4 °C overnight. Then, the beads were washed and incubated with proteinase K to remove proteins. Finally, RNA was extracted and subjected to qRT-PCR.

### Fatty acid β-oxidation (FAO) rate assay and oxygen consumption rate (OCR) measurement

For FAO rate assay, cells were incubated with unlabeled palmitate for 18 h, fresh medium containing [^3^H]-palmitate (20 μCi/mL) and fatty acid-free BSA were added, followed by further incubation for 3 h. The ^3^H_2_O in aqueous phase was collected and determined as described previously [52]. Then, scintillation solution was added, and radioactivity was measured using liquid scintillation counter (LS6500, Beckman Coulter, USA). For OCR measurement, cells were seeded in XFp Seahorse plates and performed as previously described [53].

### Mitochondrial complex I activity, NAD+/NADH ratio, and Intracellular ATP concentration measurements

Mitochondrial complex I activity, NAD+/NADH ratio, and Intracellular ATP concentration were determined using the Mitochondrial Respiratory Chain Complex I Activity Assay Kit (Solarbio, China), NAD^+^/NADH Quantification Kit (Beyotime, China), and a firefly luciferase-based ATP Assay Kit (Beyotime, China) following the manufacturers’ instructions, respectively.

### Xenograft tumor studies

All animal experiments were approved by the Animal Ethics and Experimental Committee of the Chongqing Medical University (Number: 2013-002) and performed according to the National Institutes of Health Guide for the Care and Use of Laboratory Animals. BALB/c-nude mice (females, 4–6 weeks, 16–20 g) were randomized into four groups (*n* = 5) and subjected to orthotopic injection with HGSOC cells into the right ovary fat pad. Tumor progression was monitored every two weeks by bioluminescent imaging. After sacrifice, ascitic fluid volume was measured, tumors were removed and the mass, weight, and volume were determined. Tumor tissues were then subjected to IHC staining and transmission electron microscope analysis.

### Transmission electron microscope (TEM) analysis

Tumor tissues were fixed in 2.5% glutaraldehyde, then were post-fixed with 1% osmium tetroxide in PBS for 2 h at 4 °C. After embedding in Epoxy Resin-Araldite (M) CY212 (TAAB, UK), semi-thin 2-µm-thick sections were stained with toluidine blue. Ultra-thin sections were observed using a Morgagni 268 D electron microscope (FEI Company, Italy). A number of the mitochondrial cristae was calculated manually, and the length and width of crista was measured using ImageJ software.

### Statistical analysis

Data are presented as means ± SD of three independent experiments unless otherwise indicated. Statistical analyses were performed using SPSS statistical software (Version 17.0, USA). Comparisons between two groups were evaluated by Student’s *t*-test. ANOVA was used to compare the means of more than two groups. Kaplan–Meier survival analysis and log-rank test were used to perform the survival analysis. Spearman’s correlation test was used to determine the correlation between the IHC staining intensity of Ct-OATP1B3 and IGF2BP2. *P* < 0.05 was considered statistically significant.

## Supplementary information

Supplementary Figures

Supplementary Tables

Supplementary Matierals and Methods

Full length western blots

Reproducibility checklist

## Data Availability

The data that support the findings of this study are available from the corresponding author upon reasonable request.
