# Supplementary material for: Ct-OATP1B3 promotes high-grade serous ovarian cancer metastasis by regulation of fatty acid beta-oxidation and oxidative phosphorylation
Source: Cell Death Dis. 2022 Jun 18;13(6):556. doi: 10.1038/s41419-022-05014-1 (PMC9206684; doi:10.1038/s41419-022-05014-1)
Supplement: Supplementary file 1 — Supplementary Figures [file 41419_2022_5014_MOESM1_ESM.docx]

**
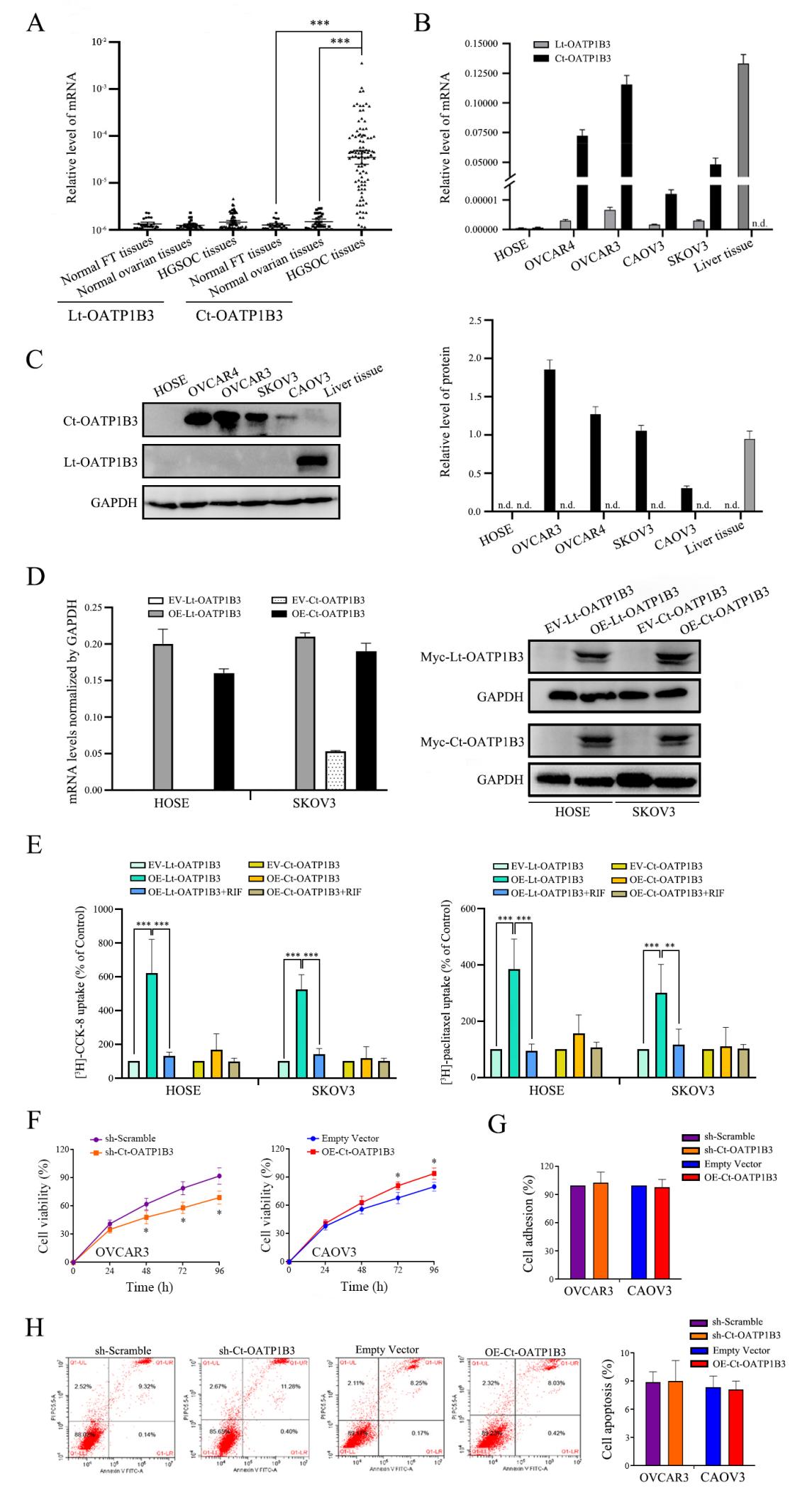
**

**Figure S1. Expression and function of Ct-OATP1B3 in HGSOC tissues and cell lines**

**A** *Lt-OATP1B3* and *Ct-OATP1B3* mRNA levels in normal fallopian tube (FT) tissues, normal ovarian tissues, as well as HGSOC tissues. **B** *Lt-OATP1B3* and *Ct-OATP1B3* mRNA levels in normal ovarian surface epithelium (HOSE) and HGSOC cell lines. **C** Lt-OATP1B3 and Ct-OATP1B3 protein levels in normal ovarian surface epithelium (HOSE) and HGSOC cell lines. **D** Establishment of stable cell lines overexpressing Myc-Lt-OATP1B3 or Myc-Ct-OATP1B3. Myc-Lt-OATP1B3 or Myc-Ct-OATP1B3 overexpression plasmid was stably transfected into HOSE and SKOV3 cells, qPCR was used to detect the expression levels of *Lt-OATP1B3* or *Ct-OATP1B3* mRNA, Western blot and anti-Myc antibody were used to detect the expression level of Myc-Lt-OATP1B3 or Myc-Ct-OATP1B3 protein. **E** Lt-OATP1B3- and Ct-OATP1B3-mediated uptake of [^3^H]-CCK-8 and [^3^H]-paclitaxel in HOSE and SKOV3 cells. **F** The influences of Ct-OATP1B3 on proliferation of HGSOC cells. **G** The influences of Ct-OATP1B3 on adhesion of HGSOC cells. **H** Cell apoptosis of HGSOC cells after knockdown or overexpression of Ct-OATP1B3. ^*^*P*<0.05, ^**^*P*<0.01, ^***^*P*<0.001.


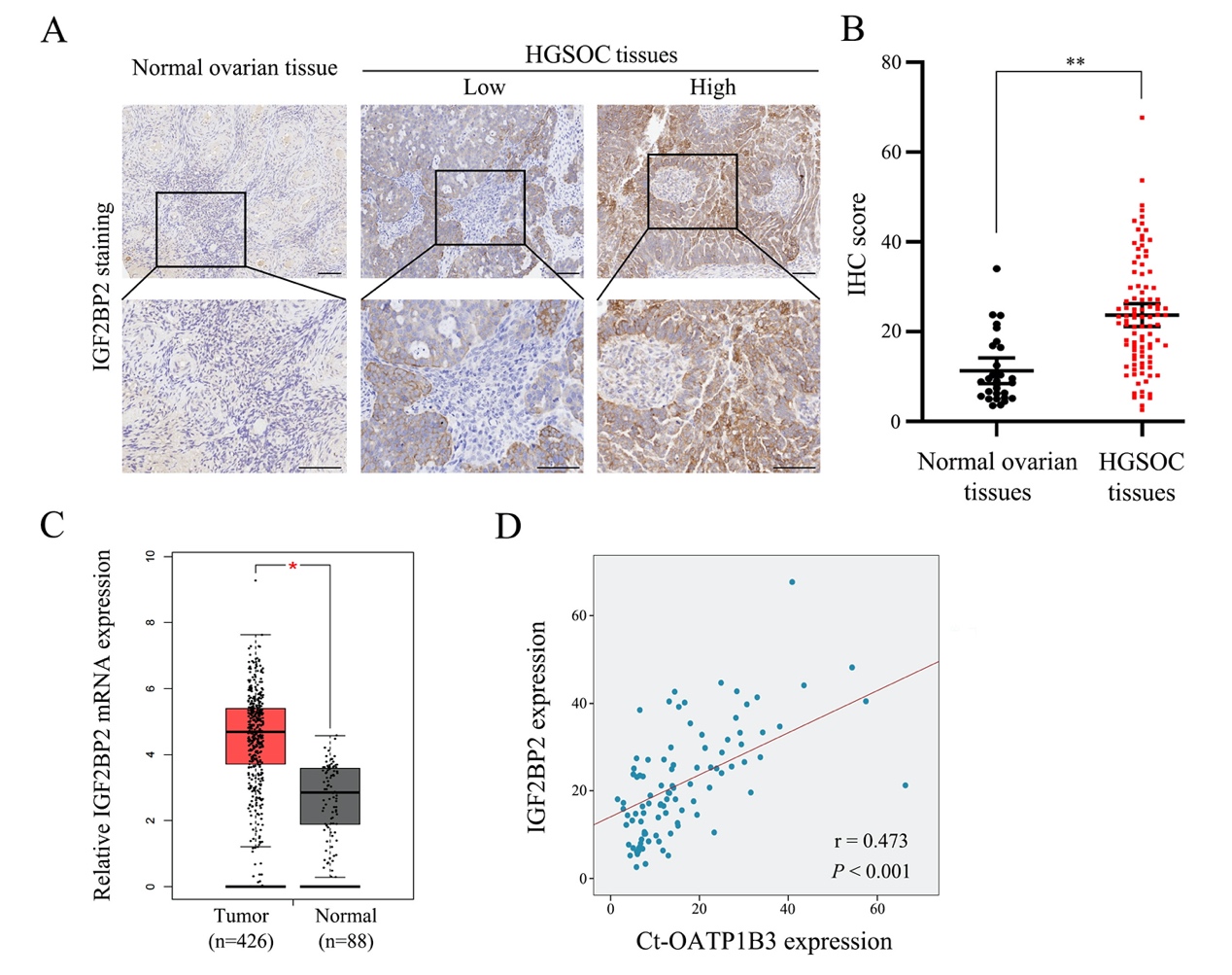


**Figure S2. The expression of IGF2BP2 is correlated with Ct-OATP1B3 in HGSOC tissues**

**A** Representative images of IGF2BP2 staining in normal ovarian epithelial sample and HGSOC tissues. Scale bar, 100μm. **B** Quantification of IGF2BP2 staining in 97 HGSOC and 29 normal ovarian tissues. **C** Higher expression of *IGF2BP2* mRNA in HGSOC samples compared with normal control in GEPIA database. **D** Correlation between Ct-OATP1B3 and IGF2BP2 staining intensity in HGSOC patients. ^*^*P*<0.05, ^**^*P*<0.01, ^***^*P*<0.001.

**
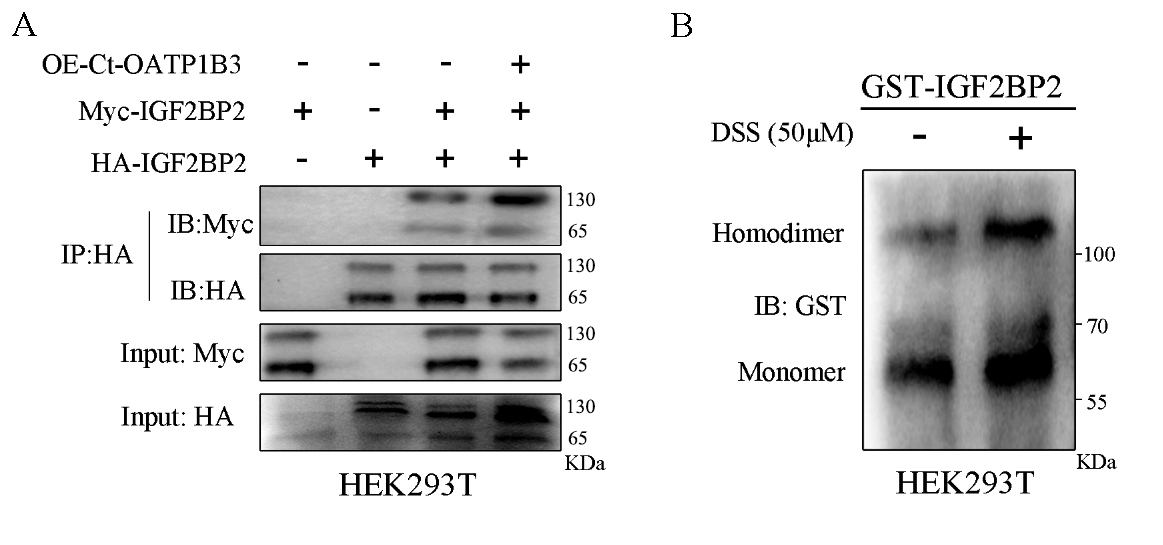
Figure S3. Homodimerization of the full-length IGF2BP2 *in vitro*.**

**A** HA-IGF2BP2 and Myc-IGF2BP2 were cotransfected into HEK-293T cells. The lysates of these cells were immunoprecipitated with an anti-HA antibody. The immunoprecipitates and input were examined by immunoblotting with the respective antibodies. **B** GST-IGF2BP2 fusion protein was incubated with the bivalent chemical cross-linker DSS. The cross-linked complexes were analyzed by Western blot using an anti-GST antibody.
