# Supplementary material for: Ct-OATP1B3 promotes high-grade serous ovarian cancer metastasis by regulation of fatty acid beta-oxidation and oxidative phosphorylation
Source: Cell Death Dis. 2022 Jun 18;13(6):556. doi: 10.1038/s41419-022-05014-1 (PMC9206684; doi:10.1038/s41419-022-05014-1)
Supplement: Supplementary file 2 — Supplementary Tables [file 41419_2022_5014_MOESM2_ESM.doc]

**Table S1**. Clinicopathological characteristics of study patients with high-grade serous ovarian cancer (n=97).

| **Variables** | **N** | **%** |
| --- | --- | --- |
| **Age at diagnosis [years] (Median, range)** | 54 | 34-75 |
| Age [years] |  |  |
| ≤50 | 36 | 37.1 |
| >50 | 61 | 62.9 |
| **Overall survival [months]** |  |  |
| Median (range) | 27.57 | 1.60-102.37 |
| Number of deaths | 30 | 30.9 |
| Number of recurrence | 36 | 37.1 |
| **Laterality** |  |  |
| Right side | 17 | 17.5 |
| Left side | 27 | 21.3 |
| Bilateral | 61 | 62.9 |
| **FIGO (%)** |  |  |
| I | 8 | 8.2 |
| II | 15 | 15.5 |
| III | 63 | 64.9 |
| IV | 11 | 11.3 |
| **Intravascular tumor thrombus (%)** |  |  |
| Yes | 31 | 32.0 |
| No | 66 | 68.0 |
| **Serum CA 125 (U/mL)** |  |  |
| <35 | 35 | 36.1 |
| ≥35 | 62 | 63.9 |
| **Chemotherapy** |  |  |
| Yes | 80 | 82.5 |
| No | 17 | 17.5 |

**Table S2**. Association of Ct-OATP1B3 expression with clinicopathologic parameters of HGSOC.

| **Variables** | **OATP1B3** | | ***P* value** |
| --- | --- | --- | --- |
|  | **Low (n=48)** | **High (n=49)** |  |
| **Age** |  |  | 1.000 |
| ≤50 | 18 (50.0%) | 18 (50.0%) |  |
| >50 | 30 (50.8%) | 31 (49.2%) |  |
| **FIGO stage** |  |  | 0.018^*^ |
| I/II | 17 (73.9%) | 6 (26.1%) |  |
| III/IV | 31 (41.9%) | 43 (58.1%) |  |
| **Intravascular tumor thrombus** |  |  | 0.083 |
| No | 31 (47.0%) | 35 (53.0%) |  |
| Yes | 14 (45.2%) | 17 (54.8%) |  |
| **CA125** |  |  | 0.162 |
| <35 U/mL | 11 (31.4%) | 24 (68.6%) |  |
| ≥35 U/mL | 29 (46.8%) | 33 (53.2%) |  |

Fisher’s exact test

**Table S3**. Univariate and multivariate analyses of factors associated with HGSOC patients’ survival (n=97).

| **Variables** | **Overall survival** | |  | **Disease-free survival** | |
| --- | --- | --- | --- | --- | --- |
|  | **HR (95% CI)** | ***P* value** |  | **HR (95% CI)** | ***P* value** |
| ***Univariate analyses*** |  |  |  |  |  |
| Age (>50 vs. ≤50) | 0.92 (0.44-1.90) | 0.81 |  | 1.20 (0.60-2.41) | 0.60 |
| FIGO stage (III/IV *vs.* I/II) | 8.00 (1.89-33.79) | 0.005^*^ |  | 6.24 (1.90-20.57) | 0.003^*^ |
| Intravascular tumor thrombus (Yes *vs.* No) | 1.29 (0.59-2.84) | 0.52 |  | 1.40 (0.70-2.81) | 0.35 |
| CA125 (≥35 U/mL *vs.* <35 U/mL) | 0.32 (0.15-0.68) | 0.003^*^ |  | 0.29 (0.15-0.58) | <0.001^*^ |
| OATP1B3 (High *vs.* Low) | 2.76 (1.26-6.04) | 0.011^*^ |  | 2.17 (1.004-3.87) | 0.039^*^ |
| ***Multivariate analyses*** |  |  |  |  |  |
| FIGO stage (III/IV *vs.* I/II)^#^ | 5.63 (1.24-25.56) | 0.031^*^ |  | 4.04 (1.14-14.26) | 0.037^*^ |
| OATP1B3 (High *vs.* Low)^§^ | 3.63 (0.72-3.67) | 0.033^*^ |  | 3.11 (0.55-2.27) | 0.045^*^ |

HR, hazard ratio; 95% CI, 95% confidence interval

^#^Adjusted for age, intravascular tumor thrombus, and CA125.

^§^Adjusted for age, FIGO stage, intravascular tumor thrombus, and CA125.

**Table S4. Primer pairs used in this study**

| **Target** | **Primer** | **Sequence 5'-3'** | **Amplicon size** |
| --- | --- | --- | --- |
| Ct-OATP1B3 | Forward | TTGGCTTGGGCTCAGAGA | 93 bp |
|  | Reverse | TGCCAAGAACATCTGCTAGTTT |  |
| Lt-OATP1B3 | Forward | GCTTTCGCAGATTAGAGGGA | 177 bp |
|  | Reverse | GCTAACTTTTTGTTGGGAAT |  |
| IGF2BP2 | Forward | AACAGGACTGTCCGTGCTAT | 154 bp |
|  | Reverse | CTCTGGATAAGAGTGATGAT |  |
| CPT1A | Forward | TGGGCTATGCGGAGGAT | 135 bp |
|  | Reverse | CCAGAAGATTTGCGGTGTT |  |
| NDUFA2 | Forward | GCAGCAAGTCGAGGAGTCG | 107 bp |
|  | Reverse | CGTTTCTCAATGAAGTCCCTGA |  |
| GAPDH | Forward | ACAACTTTGGTATCGTGGAAGG | 101 bp |
|  | Reverse | GCCATCACGCCACAGTTTC |  |
