# Supplementary material for: Ct-OATP1B3 promotes high-grade serous ovarian cancer metastasis by regulation of fatty acid beta-oxidation and oxidative phosphorylation
Source: Cell Death Dis. 2022 Jun 18;13(6):556. doi: 10.1038/s41419-022-05014-1 (PMC9206684; doi:10.1038/s41419-022-05014-1)
Supplement: Supplementary file 3 — Supplementary Matierals and Methods [file 41419_2022_5014_MOESM3_ESM.docx]

**Cell proliferation, apoptosis, and adhesion assays.**

Cell proliferation was detected using Cell Counting Kit-8 (CCK-8) method. Cells were seeded in a 96-well plate at a density of 5×10^3^ cells per well. After 24 h of incubation, cells were treated with 15 μL of CCK-8 solution (Dojindo Molecular Technologies, Japan) and incubated for a further 4 h. The absorbance was measured at 450 nm with a Hitachi F-7000 fluorescence spectrophotometer (Hitachi High-Tech, Japan). For apoptosis assay, cells were incubated with 5 mL of fluorescein isothiocyanate (FITC)-conjugated annexin V (Dojindo Molecular Technologies, Japan) and 5 mL of propidium iodide (PI) for 15 min at room temperature in the dark. Cell apoptosis rate was analyzed using a CytoFLEX flow cytometer (Beckman Coulter, USA). For the cell adhesion assay, cells were trypsinized and seeded at 1 × 10^4^ on 96-well plates that were pre-coated with 10 μg/mL fibronectin (Sigma-Aldrich, USA). After 2h incubation, culture medium was removed, and the attached cells were counted using CCK-8 assay.

**Transport assays**

HOSE and SKOV3 cells stably overexpressing Ct-OATP1B3 were seeded in poly-L-lysine coated 24-well plates, and preincubated with Krebs-Henseleit buffer (Sigma-Aldrich, USA) at 37^o^C for 15 min, then incubated in 200μL of Krebs-Henseleit buffer containing [^3^H]-CCK-8 (10nM), or [^3^H]-paclitaxel (25nM) (China Isotope & Radiation Corporation) at 37^o^C for 5 min with or without rifampin (100μM), an OATP1B3 inhibitor. The uptake reaction was terminated by adding ice-cold Krebs-Henseleit buffer followed by three washes. The cells were lysed with 0.2N NaOH and the radioactivity was measured in a liquid scintillation counter (LS6500, Beckman Coulter, USA). The substrates uptake in each well was normalized to the protein amount measured using the Pierce^TM^ BCA protein assay kit (Thermo Fisher Scientific, USA).
