# Supplementary material for: Ct-OATP1B3 promotes high-grade serous ovarian cancer metastasis by regulation of fatty acid beta-oxidation and oxidative phosphorylation
Source: Cell Death Dis. 2022 Jun 18;13(6):556. doi: 10.1038/s41419-022-05014-1 (PMC9206684; doi:10.1038/s41419-022-05014-1)
Supplement: Supplementary file 4 — Full length western blots [file 41419_2022_5014_MOESM4_ESM.docx]

**Figure 2B**

1: Plasma membrane (OVCAR3)

2: Cytoplasm (OVCAR3)

3: Plasma membrane (CAOV3)

4: Cytoplasm (CAOV3)


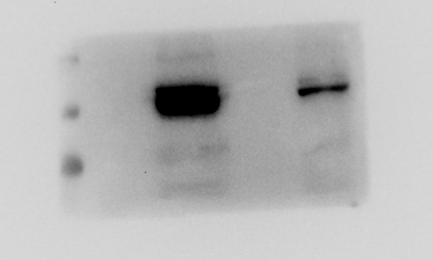
Ct-OATP1B3


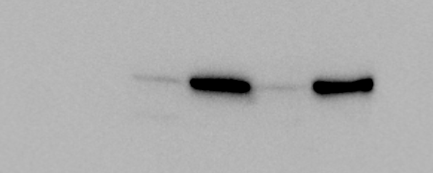
GAPDH


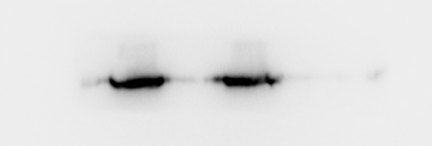
Na^+^/K^+^ ATPase

**Figure 2D**

1: sh-Scramble (OVCAR3)

2: sh-Ct-OATP1B3 (OVCAR3)

3: sh-Scramble (OVCAR3)

4: sh-Ct-OATP1B3 (OVCAR3)

5: sh-Scramble (OVCAR3)

6: sh-Ct-OATP1B3 (OVCAR3)


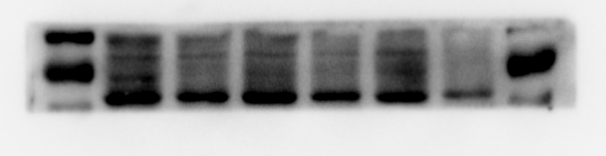
Ct-OATP1B3


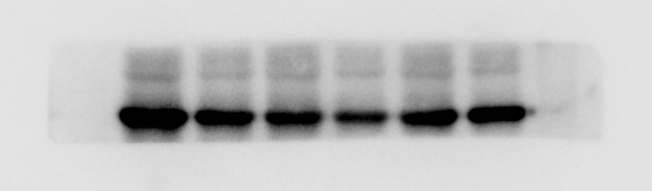
GAPDH

1: Empty vector (CAOV3)

2: OE-Ct-OATP1B3 (CAOV3)

3: Empty vector (CAOV3)

4: OE-Ct-OATP1B3 (CAOV3)

5: Empty vector (CAOV3)

6: OE-Ct-OATP1B3 (CAOV3)


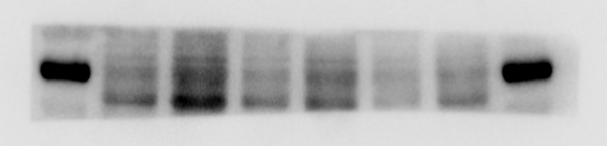
Ct-OATP1B3
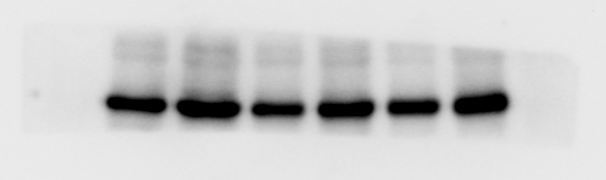
GAPDH

**Figure 3C**

1: Empty Vector; 2: OE-Myc-Ct-OATP1B3 (IgG); 3: OE-Myc-Ct-OATP1B3 (IP)

4: Empty Vector; 5: OE-Myc-Ct-OATP1B3 (IgG); 6: OE-Myc-Ct-OATP1B3 (IP)

IP: Myc


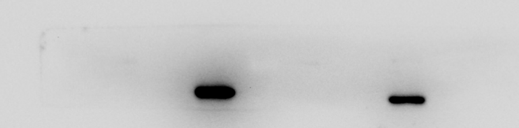
Myc


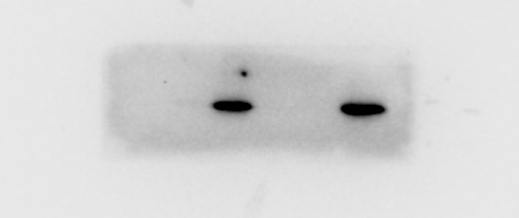
Ct-OATP1B3
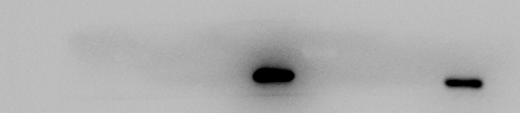
IGF2BP2

Input (IP: Myc)

1: Empty Vector; 2: OE-Myc-Ct-OATP1B3 (IgG); 3: OE-Myc-Ct-OATP1B3 (IP-1); 4: OE-Myc-Ct-OATP1B3 (IP-2)


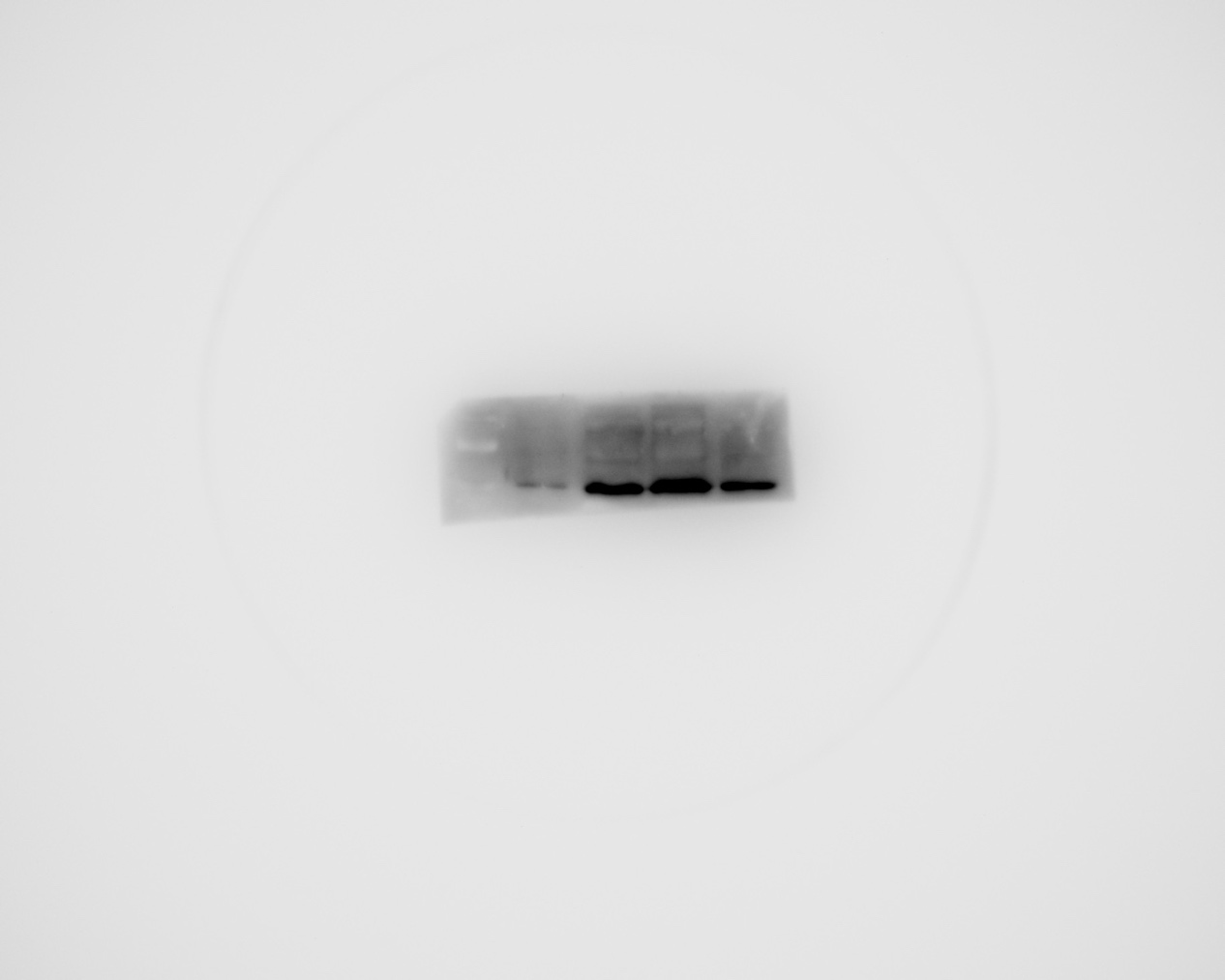
Myc

1: Empty Vector; 2: OE-Myc-Ct-OATP1B3 (IgG); 3: OE-Myc-Ct-OATP1B3 (IP)

4: Empty Vector; 5: OE-Myc-Ct-OATP1B3 (IgG); 6: OE-Myc-Ct-OATP1B3 (IP)


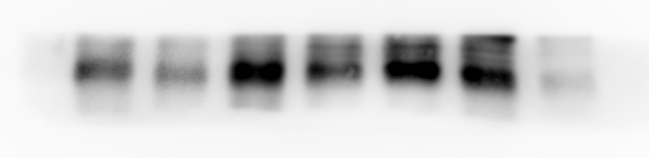
Ct-OATP1B3


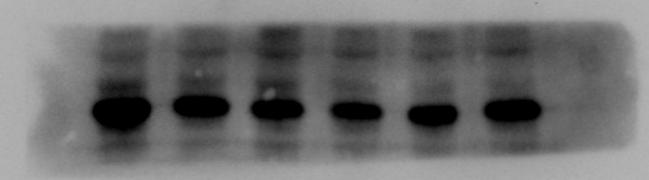
IGF2BP2

IP: IGF2BP2


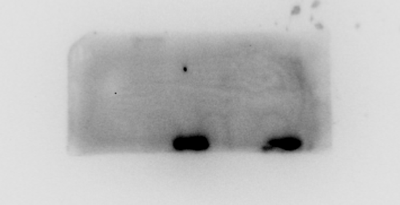
IGF2BP2


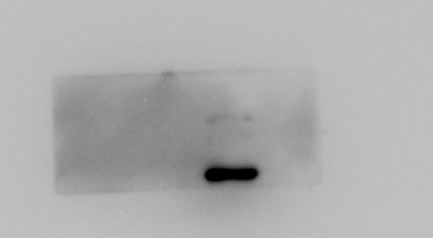
Ct-OATP1B3Myc


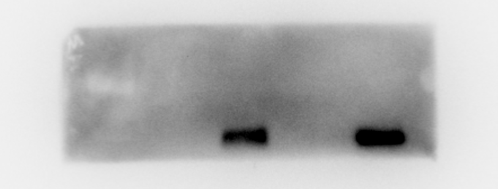
Myc

Input (IP: IGF2BP2)


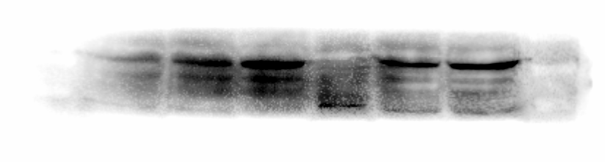
Myc


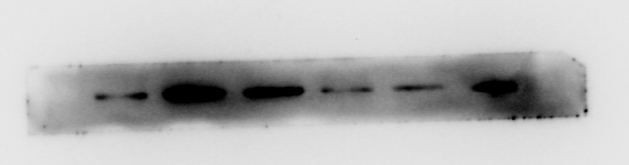
Ct-OATP1B3


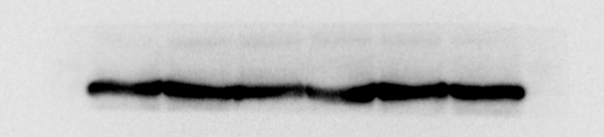
IGF2BP2

**Figure 3D**

IP: IGF2BP2

1: IgG;2: IP; 3: IgG; 4: IP


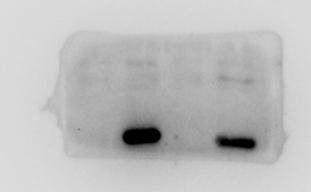
IGF2BP2


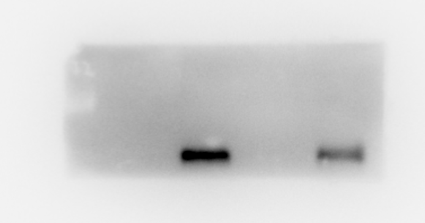
Ct-OATP1B3

Input

1: IgG;2: IP; 3: IgG; 4: IP


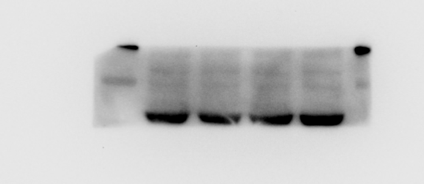
Ct-OATP1B3


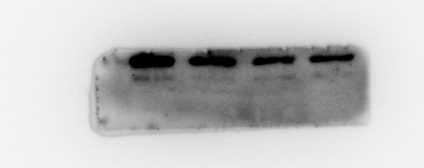
IGF2BP2

**Figure 5A**

OVCAR3

1: DSS

2: sh-Scramble

3: sh-Scramble+OE-IGF2BP2

4: sh-Scramble+si-IGF2BP2

5: sh-Ct-OATP1B3

6: sh-Ct-OATP1B3+OE-IGF2BP2


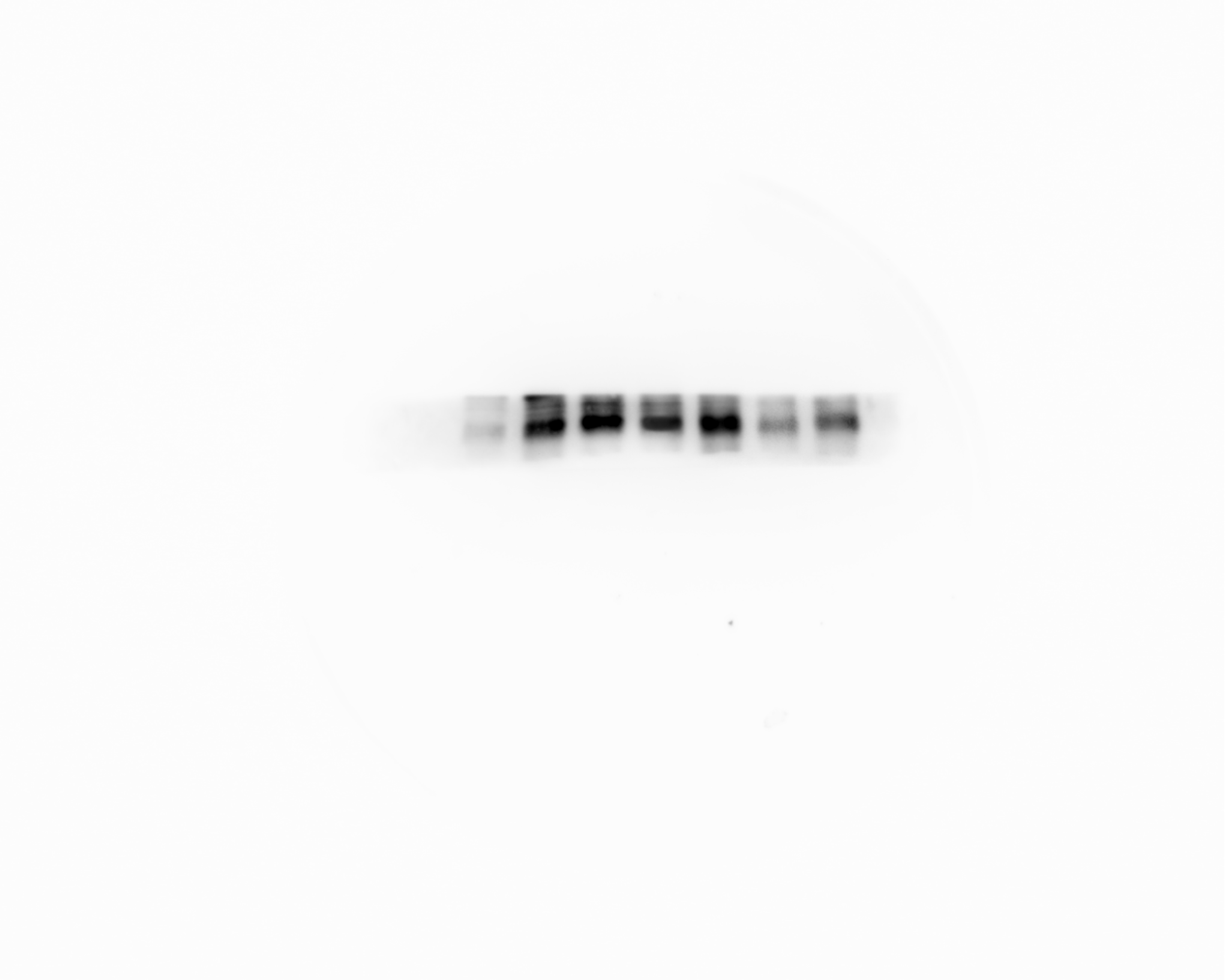


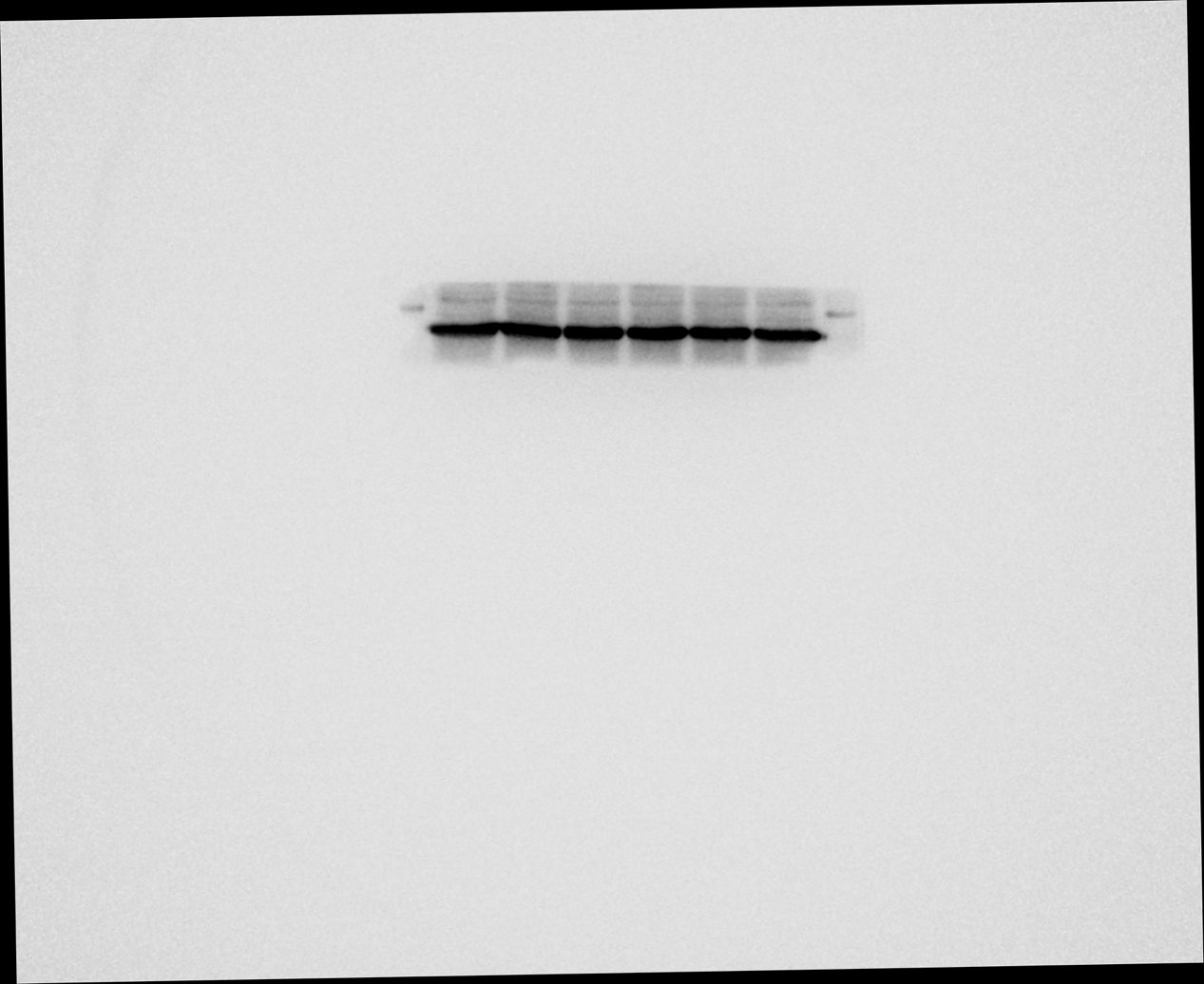


1: DSS

2: sh-Scramble

3: sh-Scramble+OE-IGF2BP2

4: sh-Scramble+si-IGF2BP2

5: sh-Ct-OATP1B3

6: sh-Ct-OATP1B3+OE-IGF2BP2

7: DSS

8: sh-Scramble

9: sh-Scramble+OE-IGF2BP2

10: sh-Scramble+si-IGF2BP2

11: sh-Ct-OATP1B3

12: sh-Ct-OATP1B3+OE-IGF2BP2


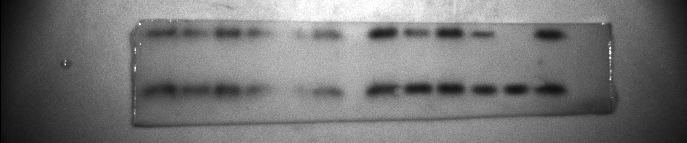


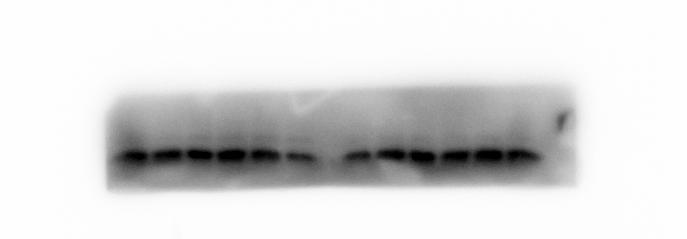


CAOV3

1: DSS (CAOV3)

2: Empty Vector (CAOV3)

3: Empty Vector+si-IGF2BP2 (CAOV3)

4: OE-Ct-OATP1B3 (CAOV3)

5: OE-Ct-OATP1B3+si-IGF2BP2 (CAOV3)


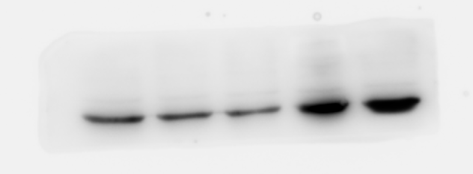
Ct-OATP1B3


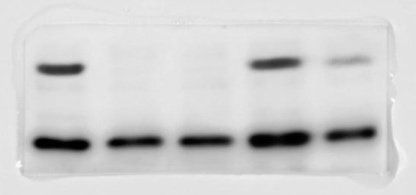
IGF2BP2


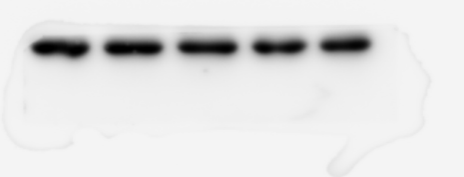
GAPDH

**Figure 5E**

1: sh-Scramble (OVCAR3)

2: sh-Ct-OATP1B3 (OVCAR3)

3: sh-Ct-OATP1B3+OE-IGF2BP2 (OVCAR3)


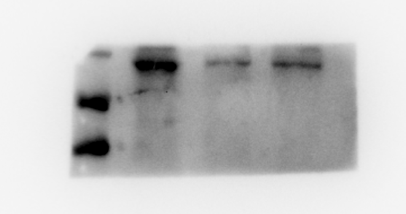
Ct-OATP1B3


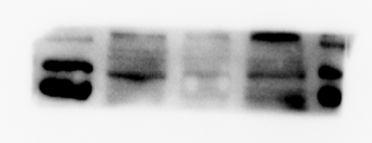
CPT1A


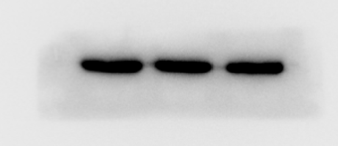
GAPDH

1: Empty Vector (CAOV3)

2: OE-Ct-OATP1B3 (CAOV3)

3: OE-Ct-OATP1B3+si-IGF2BP2 (CAOV3)

4: sh-Scramble (OVCAR3)

5: sh-Ct-OATP1B3 (OVCAR3)

6: sh-Ct-OATP1B3+OE-IGF2BP2 (OVCAR3)


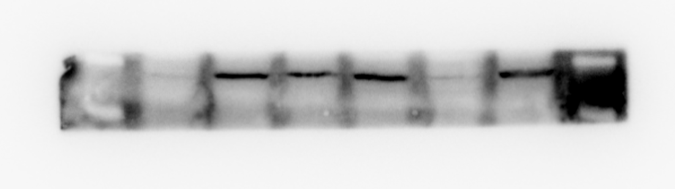
NDUFA2


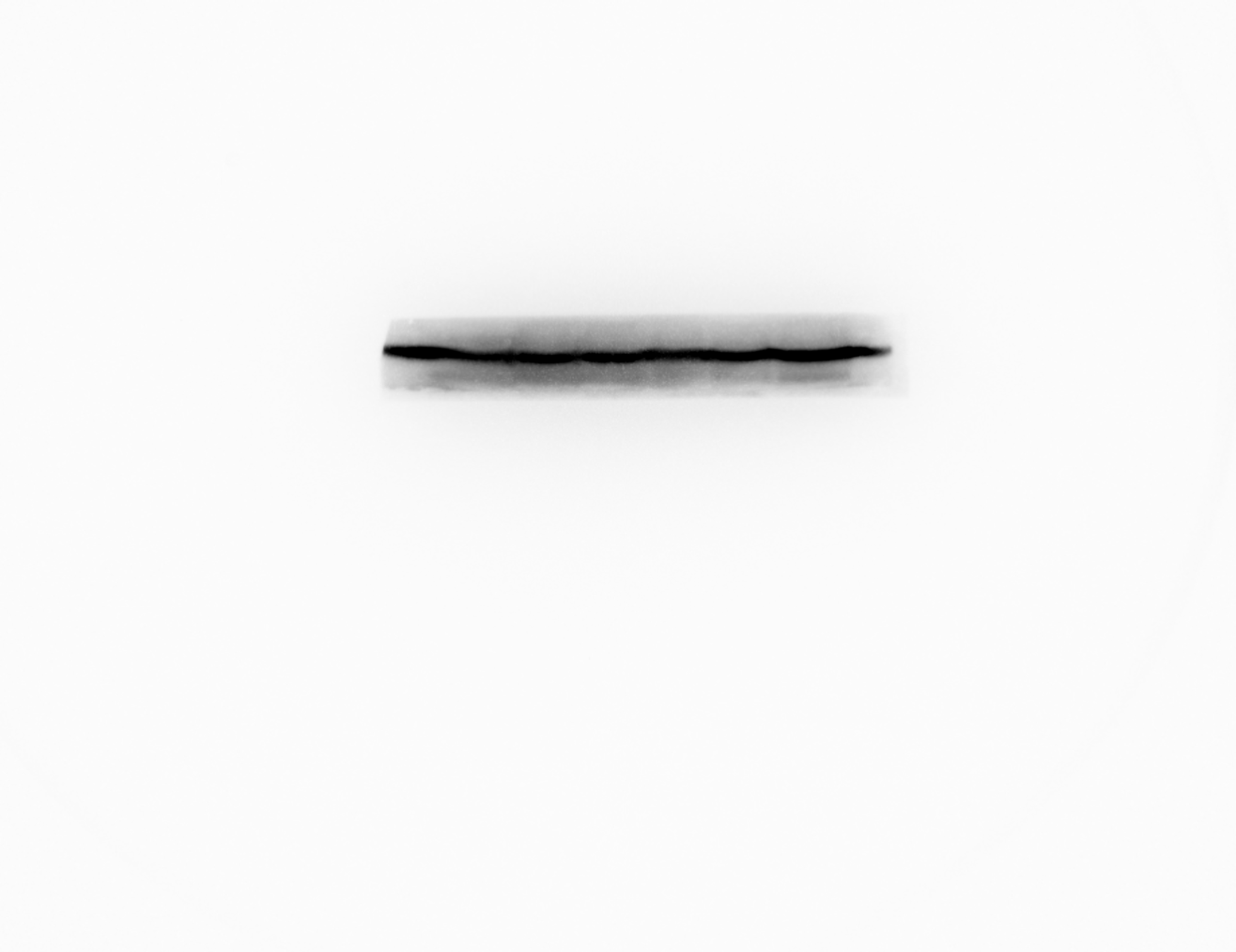
GAPDH

1: DSS (OVCAR3)；2: sh-Scramble (OVCAR3)；3: sh-Ct-OATP1B3 (OVCAR3)；4: sh-Ct-OATP1B3+OE-IGF2BP2 (OVCAR3)；5: DSS (OVCAR3)；6: sh-Scramble (OVCAR3)；7: sh-Ct-OATP1B3 (OVCAR3)；8: sh-Ct-OATP1B3+OE-IGF2BP2 (OVCAR3)


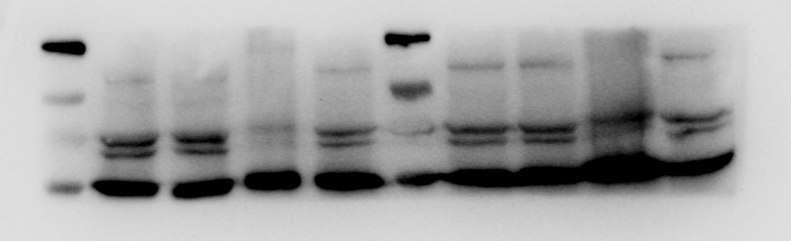
IGF2BP2 homodimer


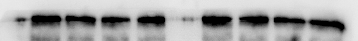
GAPDH

**Figure 5F**

1: Empty Vector (CAOV3)

2: OE-Ct-OATP1B3 (CAOV3)

3: OE-Ct-OATP1B3+si-IGF2BP2 (CAOV3)


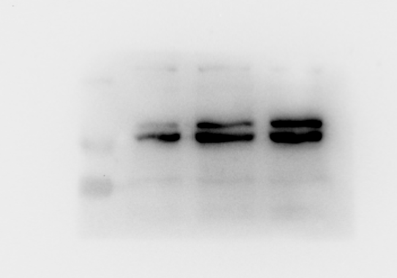
Ct-OATP1B3


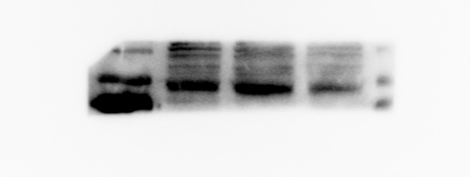
CPT1A


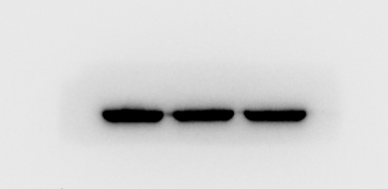
GAPDH

1: Empty Vector (CAOV3)

2: OE-Ct-OATP1B3 (CAOV3)

3: OE-Ct-OATP1B3+si-IGF2BP2 (CAOV3)

4: sh-Scramble (OVCAR3)

5: sh-Ct-OATP1B3 (OVCAR3)

6: sh-Ct-OATP1B3+OE-IGF2BP2 (OVCAR3)


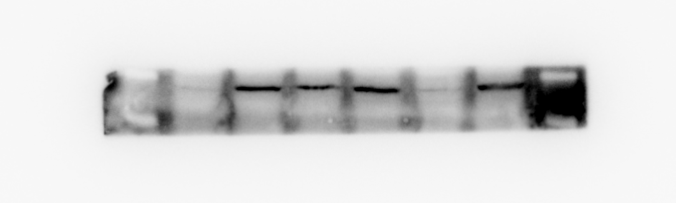
NUFA2


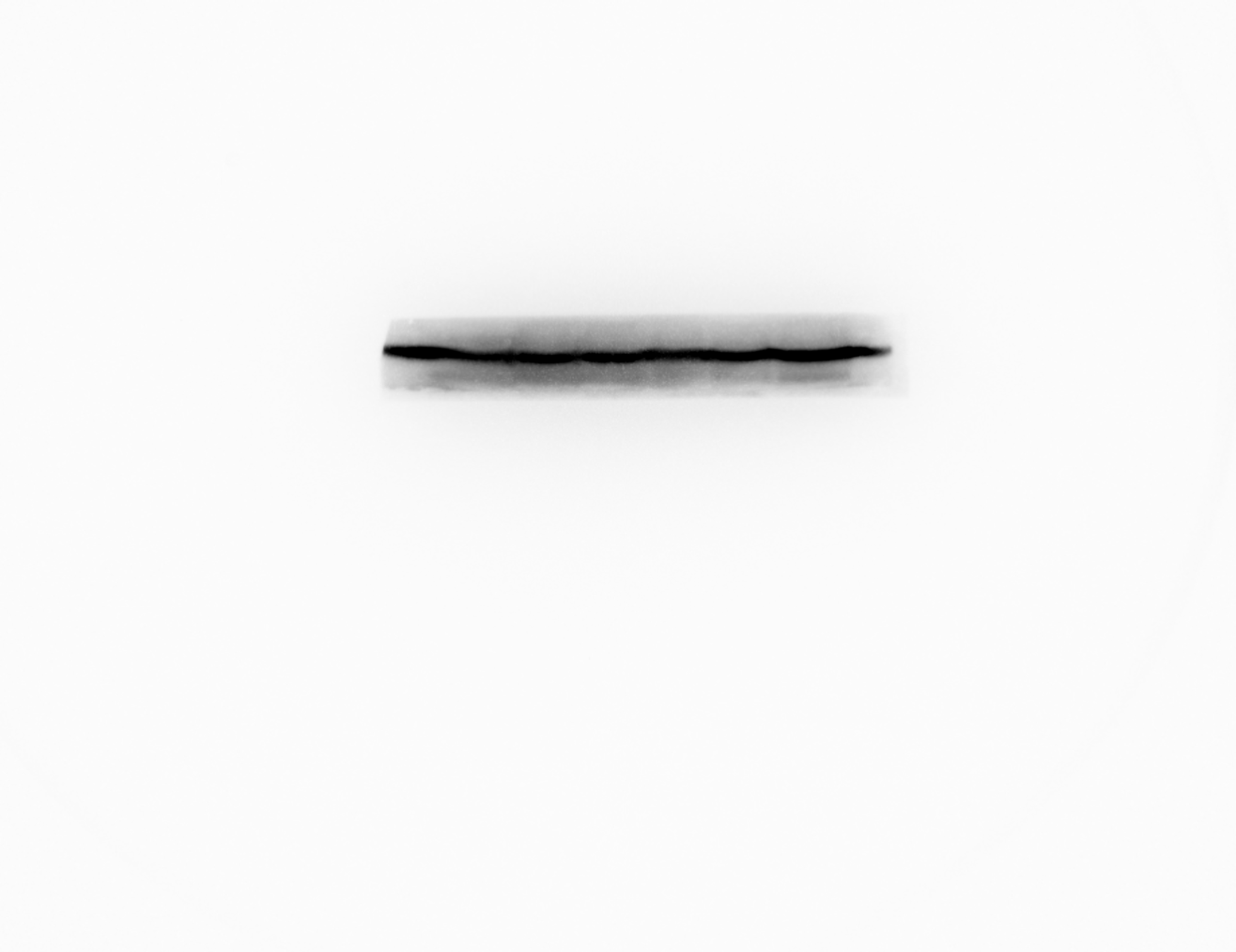
GAPDH

1: DSS (CAOV3)

2: Empty Vector (CAOV3)

3: OE-Ct-OATP1B3 (CAOV3)

4: OE-Ct-OATP1B3+si-IGF2BP2 (CAOV3)


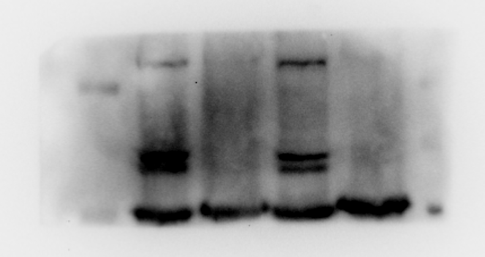
IGF2BP2 homodimer


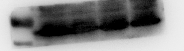
GAPDH

**Figure S1C**

1: HOSE

2: OVCAR3

3: OVCAR4

4: SKOV3

5: CAOV3

6: Liver tissue


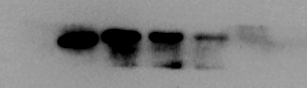
Ct-OATP1B3


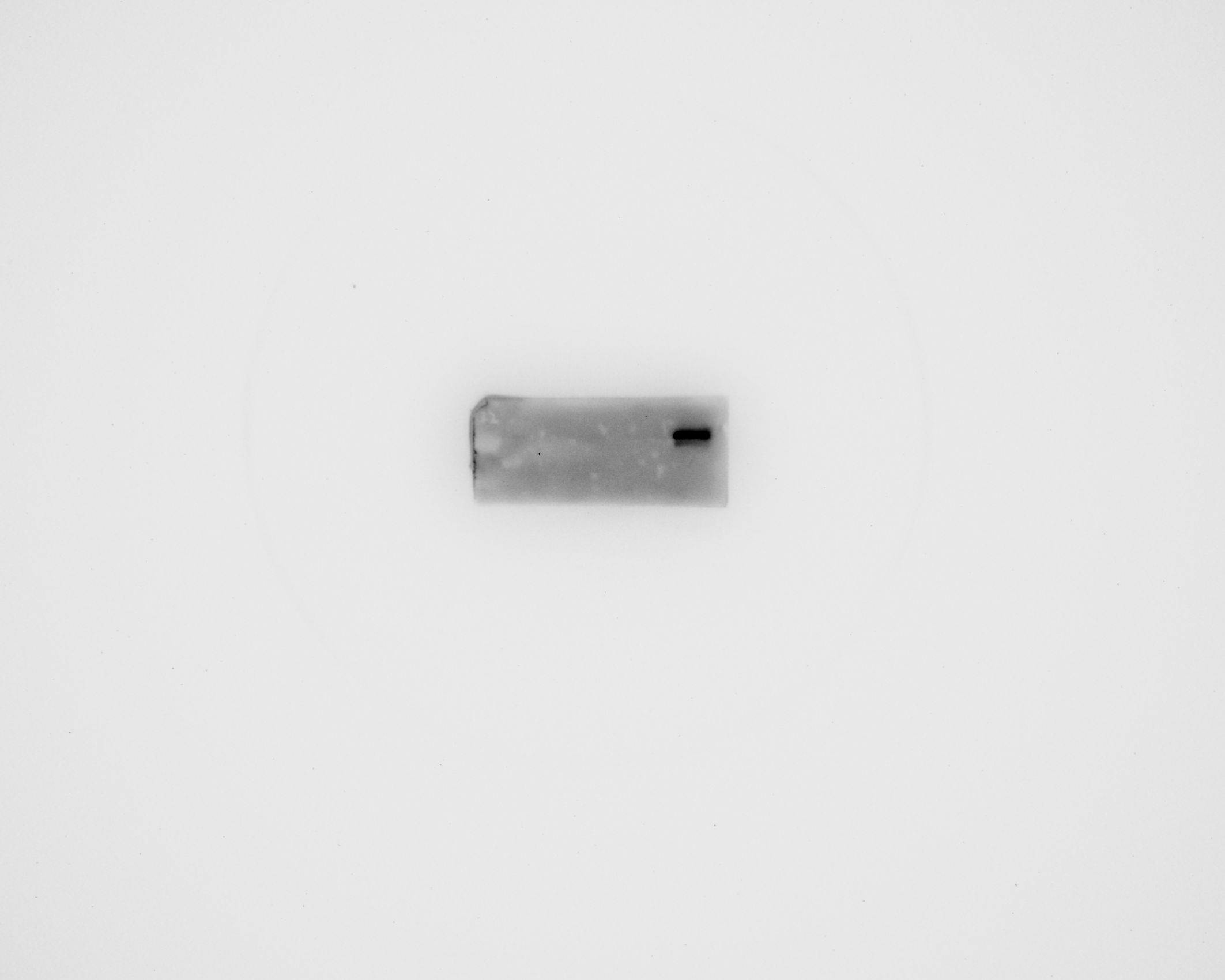
Lt-OATP1B3


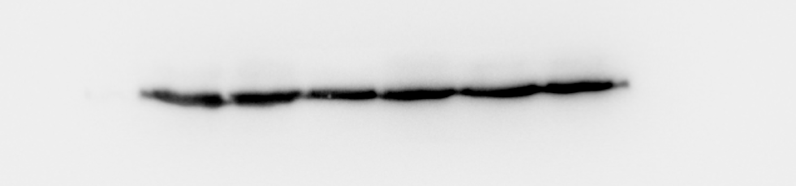
GAPDH

**Figure S1D**

1: EV-Lt-OATP1B3 (HOSE)

2: OE-Lt-OATP1B3 (HOSE)

3: EV-Ct-OATP1B3 (SKOV3)

4: OE-Ct-OATP1B3 (SKOV3)


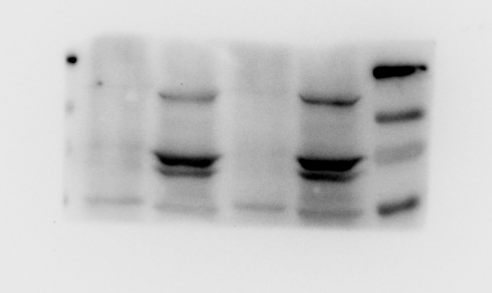
myc-Lt-OATP1B3


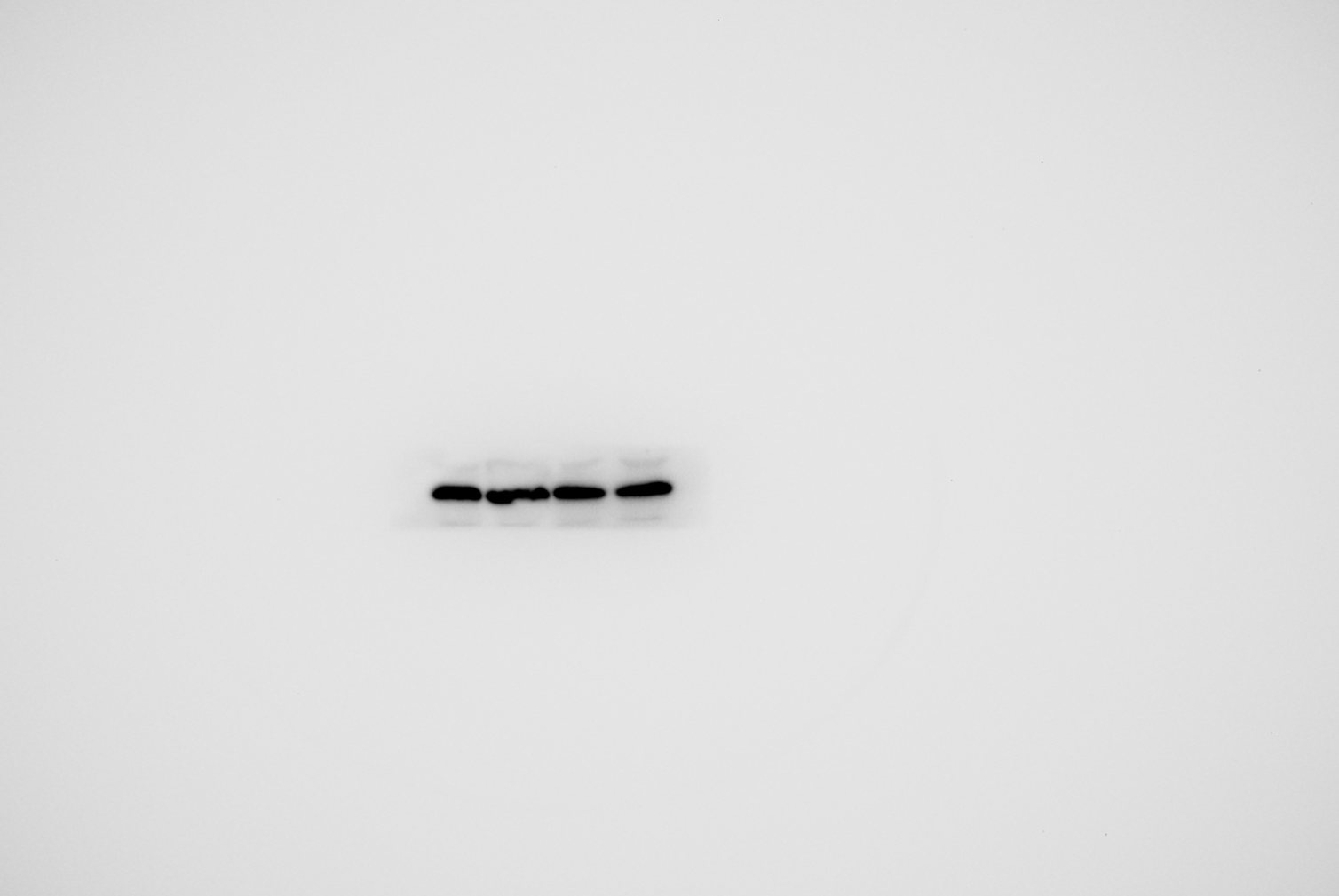
GAPDH


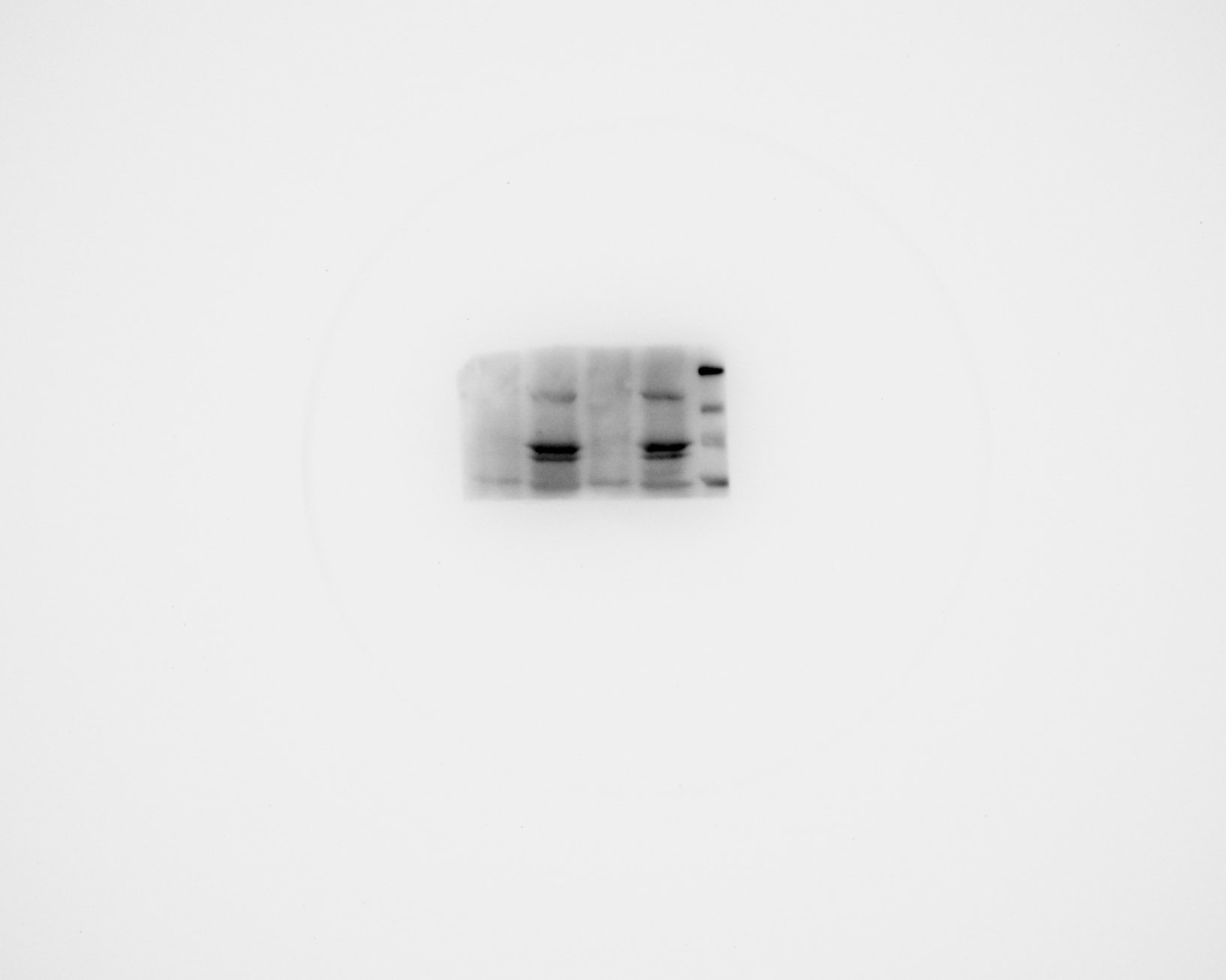
myc-Ct-OATP1B3


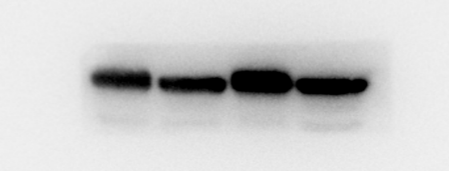
GAPDH

**Figure S3A**

IP：HA

1: Myc-IGF2BP2 (HEK293T)

2: HA-IGF2BP2 (HEK293T)

3: Myc-IGF2BP2+ HA-IGF2BP2 (HEK293T)

4: OE-Ct-OATP1B3+ Myc-IGF2BP2+ HA-IGF2BP2 (HEK293T)


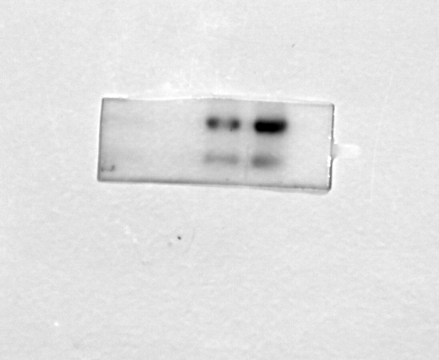
Myc


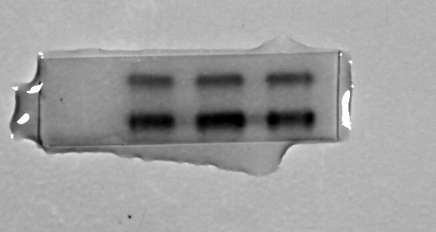
HA

Input

4: Myc-IGF2BP2 (HEK293T)

5: HA-IGF2BP2 (HEK293T)

6: Myc-IGF2BP2+ HA-IGF2BP2 (HEK293T)

7: OE-Ct-OATP1B3+ Myc-IGF2BP2+ HA-IGF2BP2 (HEK293T)


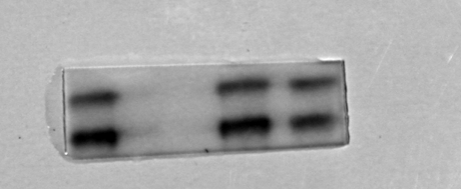
Myc


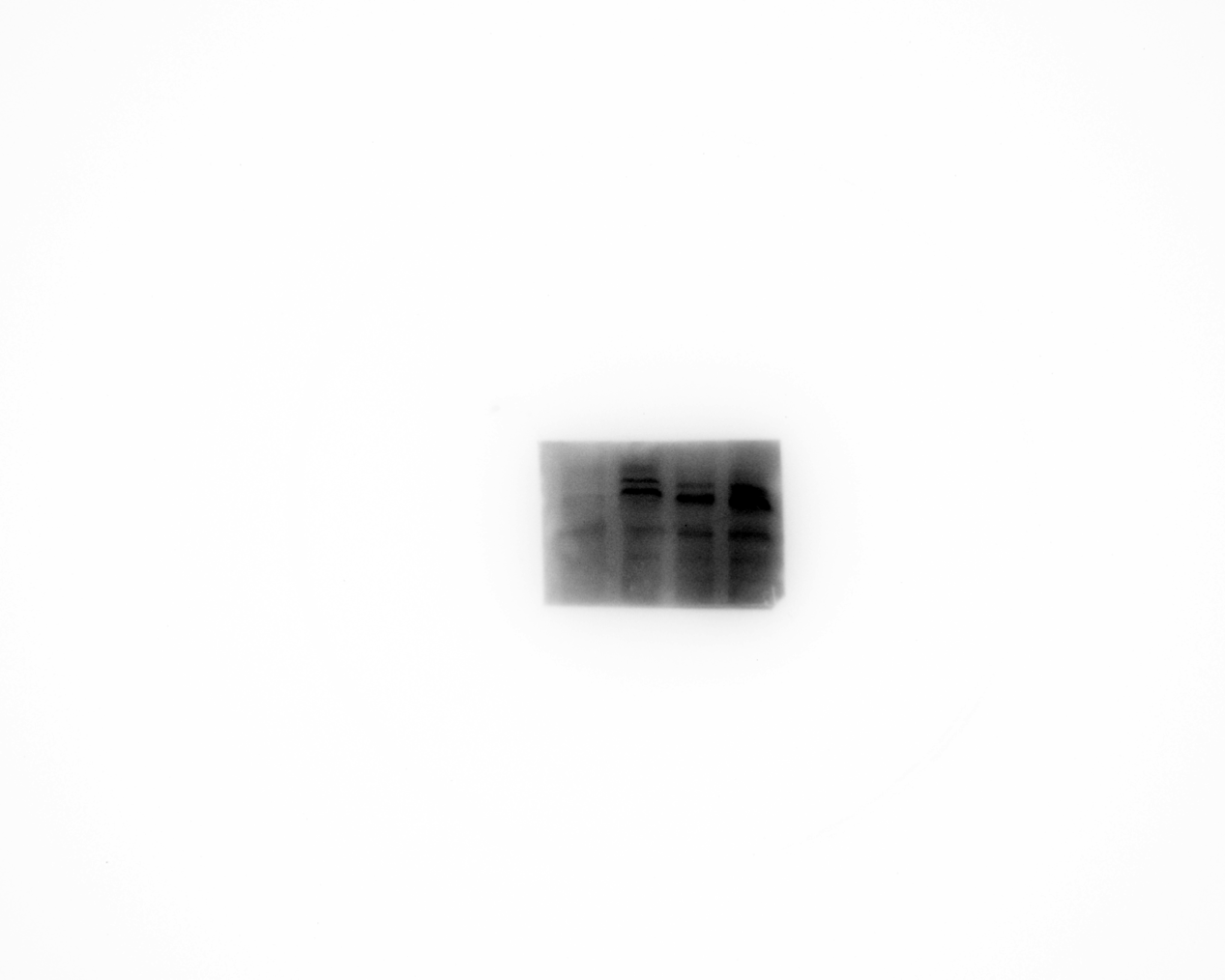
HA

**Figure S3B**

1: GST-IGF2BP2 (2μg) (HEK293T)

2: GST-IGF2BP2 (2μg) + 50μM DSS (HEK293T)

3: GST-IGF2BP2 (1μg) (HEK293T)

4: GST-IGF2BP2 (1μg) + 50μM DSS (HEK293T)


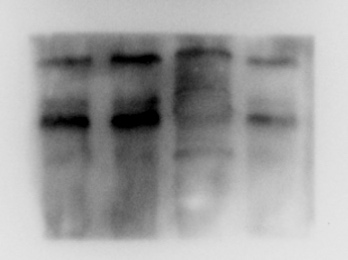
GST
